# Alpha-defensins increase NTHi binding but not engulfment by the macrophages enhancing airway inflammation in Alpha-1 antitrypsin deficiency

**DOI:** 10.3389/fimmu.2025.1543729

**Published:** 2025-02-12

**Authors:** Jungnam Lee, Naweed Mohammad, Kyudong Han, Tammy Flagg-Dowie, Maria Magallon, Mark L. Brantly, Karina A. Serban

**Affiliations:** ^1^ Division of Pulmonary, Critical Care and Sleep Medicine, University of Florida, Gainesville, FL, United States; ^2^ Department of Microbiology, College of Bio-convergence, Dankook University, Cheonan, Republic of Korea; ^3^ Center for Bio-Medical Engineering Core Facility, Dankook University, Cheonan, Republic of Korea; ^4^ Department of Medicine, Division of Pulmonary, Critical Care, and Sleep Medicine, National Jewish Health, Denver, CO, United States

**Keywords:** alpha defensins, alpha 1 antitrypsin (AAT), AAT deficiency (AATD), neutrophils, macrophages, cytokines, phagocytosis

## Abstract

Neutrophilic inflammation and a high level of free α-defensins are main features of chronic airway inflammation in alpha-1 antitrypsin-deficient (AATD) individuals. Despite the antimicrobial activities of α-defensins by direct bacterial killing and by modulation of immune responses, AATD individuals are paradoxically burdened by recurrent exacerbation triggered by bacterial infections, frequently with nontypeable *Haemophilus influenzae* (NTHi). Previous studies demonstrated that high, rather than low α-defensin level could modulate the local pro-inflammatory milieu of bronchial epithelial cells and macrophages promoting chronic inflammation and lower pathogen phagocytosis. IgG-mediated phagocytosis and NTHi adherence, engulfment and phagocytosis were measured in human alveolar macrophages and monocyte-derived macrophages (MDM) isolated from patients with AATD and from healthy individuals. A high concentration of free α-defensins induced NTHi adherence to MDMs but decreased IgG-mediated phagocytosis by MDMs. The decreased phagocytosis was associated with TLR4 activation, downstream signaling via NF-κB p65 and marked increased secretion of inflammatory cytokines, CXCL8, IL-1b, and TNFα by the α-defensin-treated and NTHi-infected MDMs. Exogenous AAT treatment and TLR4 inhibitor decreased TNFα expression in α-defensin-treated cells. Dampening the downstream effects of a high concentration of α-defensins may render AAT and TLR4 inhibitors as potential therapies to decrease NTHi colonization and increase its clearance by phagocytosis in AATD individuals.

## Introduction

The concentration of free α-defensins in the epithelial lining fluid (ELF) of healthy control subjects is less than 30nM, but it is, on average, 2000nM in that of AAT-deficient (AATD) individuals (1). The concentration of α-defensins is further increased to 6000nM in the ELF of AATD individuals with more severe lung diseases (2). Human defensins, small cationic peptides of ~30 amino acids, are divided into two subfamilies of α- and β-defensins based on their disulfide linkages. They are produced by neutrophils and sequestered within the azurophilic granules of neutrophils (3, 4). α-defensin-containing granules normally undergo restricted secretion and are fused with phagolysosomes, where high concentrations of α-defensins directly kill phagocytosed microorganisms.

At low concentrations, α-defensins are beneficial to host cells by promoting the clearance of phagocytosing pathogens, while at high concentrations, they are toxic to host cells by inducing membrane blebbing and lysis (5). In addition, they are able to induce the expression of proinflammatory cytokines. α-defensins at concentrations exceeding 10 μg/ml increase the expression levels of CXCL5, CXCL8, IL-6, and IL-1β in epithelial cells. At a concentration of 20 μg/ml, α-defensins are cytotoxic, reducing the viability of lung epithelial cells by 30% (6–8). The microbicidal concentration of α-defensins ranges from 1 to 100 μg/ml (6, 7, 9). Accumulating data suggest that TNFα is essential in the pathogenesis of AATD-associated lung diseases (10). Consistent with the expression of TNFα, the level of activated NF-κB was found to be higher in α-defensin-treated and NTHi-infected MDMs than NTHi-infected MDMs incubated without α-defensins. TLR4 is an upstream molecule of NF-κB, and it is known that NTHi activates TLR4 (11, 12).

The antimicrobial activity of α-defensins could be abolished by their microenvironment conditions such as a high concentration of salt (13, 14). Patients with cystic fibrosis (CF) are characterized by the abundance of alveolar neutrophils and a high concentration of α-defensins, both of which are supposed to suppress bacterial infection by killing and phagocytosing bacteria. Nonetheless, severe bacterial infections persist in the lung of the CF patients (15). It is not clear whether α-defensins are bactericidal when they are released from neutrophils into the alveolar lumen. When α-defensins were administered in mice, the bacterial number in experimental infections in mice was significantly reduced. However, α-defensins was not able to reduce the number of the infected bacteria in leukocytopenia mice. This suggests that local phagocyte and lymphocyte accumulation might be essential for the antibacterial effect of α-defensins (16), supporting that the environmental condition of α-defensins is important for their antimicrobial activity. The effect of α-defensins on bacterial infection in AATD has not been investigated, especially looking at the extracellular role of α-defensins on clearing nontypeable *Haemophilus influenzae* (NTHi), a prevalent pathogen of AATD infectious exacerbation.

In AATD, airway macrophages are the primary phagocytes to clear inhaled pathogens via opsonin-mediated and scavenger receptor-mediatd phagocytosis in the lung (17). It was previously reported that the phagocytic ability of macrophages is impaired in the lung of smokers and AATD individuals, and the study suggested that AAT polymer might reduce the phagocytic ability of alveolar macrophages in AATD individuals (17–19). Moreover, our recent study on α-defensins found that α-defensins suppress the phagocytic ability of macrophages by inhibiting cell motility and inhibiting the expression of pattern recognition receptors, CD163 and CD206 (20). These published data pose the question whether a high concentration of α-defensins could be responsible for the reduced phagocytic ability of alveolar macrophages in AATD individuals.

Nontypeable *Haemophilus influenzae* (NTHi) are facultative, anaerobic, Gram-negative coccobacilli (21). They are highly prevalent and pathogenic in various important lower respiratory conditions including chronic obstructive pulmonary disease (COPD), cystic fibrosis, bronchiectasis, and pneumonia (22, 23). It is not clear why NTHi is a colonizer in the upper respiratory tract but pathogenic in the lower respiratory tract. It was previously reported that α-defensins enhance the binding ability of NTHi to lung epithelial cells (13). Although AAT is a protease inhibitor that regulates the proteolytic effects of neutrophil-derived serine proteases, including neutrophil elastase, cathepsin G, and thrombin, it was reported that AAT reduces the α-defensin cytotoxicity (24). Whether free α-defensins coordinate NTHi binding, uptake, and phagocytosis to promote airway colonization in AATD could provide new therapeutic avenues to decrease infectious exacerbations and chronic inflammation in this population.

In this study, we used complementary approaches to investigate the mechanisms by which a high concentration of free α-defensins impair NTHi phagocytosis by macrophages, hence promoting NTHi colonization in the airway of AATD individuals. We found that a high concentration of α-defensins induces NTHi adherence to MDMs, decreases IgG-mediated phagocytosis by infected monocyte-derived macrophages (MDMs), activates TLR4/NF-kB p65 signaling and increases the expression of inflammatory cytokines, CXCL8, IL-1b, and TNFα in MDMs. Moreover, we demonstrated that exogenous AAT supplementation and TLR4 inhibition can decrease the TNFα expression increased by α-defensins in NTHi-infected MDMs. This study highlights a novel mechanism by which α-defensins cause uncontrolled inflammatory cytokine production and exacerbate NTHi infection in the airways of AATD individuals.

## Materials and methods

### Human samples

BAL fluid was obtained from healthy individuals after informed consent (University of Florida IRB201501133) and AATD individuals with COPD (University of Florida IRB20140928), using the protocol previously described (25, 26). The characteristics of the individuals are shown in **Table 1**. None of the AATD individuals were on AAT augmentation therapy for six weeks preceding the BAL procedure. The concentration of α-defensins was measured in the BAL fluid samples. We corrected for the variability of BAL fluid return, where despite using similar instilled saline volumes, the individuals with advanced airway obstruction had lower volume return, by using the ratio of urea concentration in the BAL fluid to that in the serum to calculate the volume of epithelial lining fluid (ELF) recovered after each BAL procedure. We have used the following formulas: Urea_BAL/_Urea_plasma_ X Vol _BAL_ = ELF and [sCX3CL1] X Vol_BAL/_ELF = sCX3CL1_ELF_ (27).

**Table 1:** Characteristics of controls and AATD individuals used for BAL samples.

Characteristic	PiMM (n=8)	PiZZ (n=16)	P-value
Age	42.3 ± 13.2	56.9 ± 7.9	0.017
Gender (M/F)	5/3	3/15	N/A
Current smoker	No	No	N/A
FEV1% predicted	103.9 ± 17.6	81.3 ± 18.1	0.074
AAT (nM)	2523.8 ± 1108.7	256.3 ± 191	0.000
Neutrophil (%)	1.2 ± 0.6	20.6 ± 21.8	0.003
Macrophage (%)	91.5 ± 3.6	73.2 ± 22.1	0.007
Lymphocyte (%)	7.3 ± 3.6	5.4 ± 6.1	0.419
Eosinophil (%)	0 ± 0	0.7 ± 1.1	0.023

Definition of abbreviations: PiMM, individuals homozygous for normal PiM allele; PiZZ, individuals homozygous for mutant PiZ allele; N/A, not applicable; FEV1, forced expiratory volume in one second. Data are presented as mean ± standard deviation (SD).

### Monocyte isolation and macrophage differentiation

Peripheral blood mononuclear cells (PBMCs) were isolated either from Leukopaks (obtained from LifeSouth Community Blood Center, Gainesville, FL) or blood samples of outpatient volunteers (University of Florida Institutional Review Board protocol 2015-01051), using Ficoll-gradient centrifugation. Monocytes were purified from PBMCs using a monocyte enrichment kit (Stemcell Technology, Vancouver) following the manufacturer’s instruction. Monocytes were plated in 12-well plates at 300,000 cells per well and incubated in macrophage differentiation media (RPMI 1640 containing 10% FBS, 100 units/mL penicillin, 100 μg/mL streptomycin, 250 ng/mL amphotericin B, recombinant human GM-CSF [0.5 ng/mL], and recombinant human M-CSF [5 ng/mL]) for 7 days. Supplemental medium (50% of the volume in each well) was added every 3 days after removing half of the old media (28), and the differentiated MDMs were incubated with α-defensins of HNP1 and HNP2 (AnaSpec, Fremont) on day 7 in serum-free media. MDMs were harvested for RNA extraction using the Qiagen RNeasy kit (Qiagen, Hilden).

### Phagocytosis of IgG-coated bead by primary alveolar macrophages and MDMs

Primary human alveolar macrophages were obtained from the BAL fluid of healthy subjects or AATD individuals under National Jewish Health/BRANY approved IRB protocols (HS-572 and HS-3401-528). Macrophages were cultured in RPMI-1640 supplemented with 1% non-essential amino-acids, 2% sodium pyruvate, 20mM Hepes, and penicillin (100 U/ml). Phagocytosis assay was performed by co-incubation of the macrophages with phagocytic IgG-coated beads for 1 hour at 37°C at 1:5 ratio. The phagocytic targets were fluorescently labeled latex beads (Sigma, Saint Louis, MO, USA) coated with 1% BSA for 1 hour at 4°C and then incubated with rabbit anti-bovine albumin antibody at a dilution of 1:500 at 37°C for 30 minutes. At the end of 1 hour co-incubation with phagocytic targets, alveolar macrophages were collected in flow cytometry tubes, and the engulfment of the latex beads by alveolar macrophages was measured by flow cytometry. Monocytes were plated in 8-well slides at 100,000 cells per well and incubated in macrophage differentiation media for 7 days. MDMs were incubated with or without α-defensins in serum free media for 16 hours, and then incubated with IgG-FITC-coated latex beads (Cayman chemical, Ann Arbor) at a dilution of 1:500. After 30 minutes of the incubation, cells were incubated with trypan blue provided in the phagocytosis assay kit for 1 minute to quench the green fluorescence of the beads attaching to the cell surface. Cells were washed with PBS three times and fixed in 4% paraformaldehyde for 20 minutes. The fixed cells were mounted on the slide using ProLong Glass Antifade Mountant with NucBlue (Invitrogen, Waltham). MDMs phagocytosing the IgG-FITC complex were visualized using a fluorescence microscope (BZ-X700, Keyence, Osaka).

### Non-typable hemophilus influenzae culture

NTHi (ATCC 53600) were cultured in Brain Heart Infusion media supplemented with hemin (10 μg/ml) and β-Nicotinamide adenine dinucleotide hydrate (10 μg/ml) overnight at 37°C in 5% CO2. The optical density at 600nm of the bacteria culture was adjusted to 1 (approximately 10^9^ CFU/ml) (29). MDMs were infected with NTHi at a multiplicity of infection (MOI) of 10. To estimate the number of cell-associated bacteria, MDMs were incubated with NTHi for three hours. After the incubation, the cells were washed with PBS six times and lysed with distilled water for twenty minutes. Serial dilutions of cell lysates were spread on chocolate agar plates (Anaerobe Systems, Morgan Hill), and the plates were incubated overnight at 37°C in 5% CO2. The number of colony forming units (CFU) was counted per sample. To examine the effect of α-defensins on dead bacteria, NTHi were incubated at 60°C for 30 minutes. The bacterial solution was incubated on chocolate agar plates (Anaerobe Systems, Morgan Hill) overnight, and it was confirmed that all bacteria were killed by the incubation.

### Antibiotic protection assay

MDM controls and the α-defensin-treated MDMs were incubated with NTHi for three hours. Cells were washed with new macrophage differentiation media without antibiotics and incubated with gentamicin (100 μg/ml) for 30 minutes. Cell supernatants were collected and used as control to confirm the extracellular bacterial killing. MDMs were then lysed with distilled water for twenty minutes, and the number of CFU was enumerated by serial dilution of lysates in PBS and plating them on chocolate agar plates (Anaerobe Systems, Morgan Hill).

### SYTOX green nucleic acid stain

Monocytes were plated in 8-well slides at 100,000 cells per well and incubated in macrophage differentiation media for 7 days. MDMs were incubated with or without α-defensins in serum-free media for 16 hours. MDM control and α-defensin-treated MDMs were incubated with NTHi at MOI 10 in macrophage differentiation media without antibiotics for one hour. Unbound NTHi were washed with new media without antibiotics, and MDMs were incubated with gentamicin (100 μg/ml) for 30 minutes. Then, the gentamicin-containing media was replaced with new media containing 5 μM of SYTOX Green Nucleic Acid Stain, and cells were incubated in the new media for 5 minutes. After the incubation, cells were washed with PBS one time and fixed in 4% paraformaldehyde for 20 minutes. The fixed cells were mounted on the slide using ProLong Glass Antifade Mountant with NucBlue (Invitrogen, Waltham). NTHi bound to MDMs were visualized using a fluorescence microscope (BZ-X700, Keyence, Osaka).

### Gene expression by qRT-PCR

Total RNAs (500 ng) extracted from MDMs were reverse transcribed using SuperScript^®^ VILO Master Mix (Invitrogen, Carlsbad) according to the manufacturer’s instruction. Quantification of PCR products was performed by 7500 Fast Real-time PCR (Applied Biosystems, Foster City). TaqMan™ Fast Advanced Master Mix for qPCR (Applied Biosystems, Foster City) was used to produce fluorescence-labeled PCR products and to monitor increasing fluorescence during repetitive cycling of the amplification reaction. TaqMan probes/primers specific for CXCL8, IL-1b, and TNFα genes, and for the 18S rRNA gene, as the internal control, were used in the real-time PCR reaction. Expression levels of the genes were obtained using the classical 2^(-ΔΔCt) method.

### ELISA

TNFα was measured in conditioned media of MDMs using a sandwich enzyme-linked immunosorbent assay (ELISA). MDMs were incubated with α-defensins for 16 hours, and the α-defensin-treated MDMs were incubated with NTHi for three hours. Conditioned media were collected from the MDM culture, and the concentration of TNFα was measured in the conditioned media by ELISA (Abcam, Cambridge), following the manufacturer’s instruction. The concentrations of α-defensins were measured in bronchoalveolar lavage (BAL) fluids of control and AATD individuals using ELISA (R&D Systems, Minneapolis).

### Western blot analysis

Total proteins were extracted from MDMs using RIPA lysis buffer (Cell Signaling Technology, Danvers) with 0.1% SDS, protease inhibitors and phosphatase inhibitors. The protein concentration of each sample was measured using a standard Bradford assay (BioRad, Hercules) and equal amounts of protein samples were loaded onto an SDS polyacrylamide gel (BioRad, Hercules). After gel electrophoresis, the proteins were transferred onto a nitrocellulose membrane using a wet-transfer system, and the membrane was blocked in Tris-buffered saline with 0.1% Tween 20 (TBST) containing 5% nonfat dry milk. When detecting the phosphorylated form of any target proteins, Tris-buffered saline with 0.1% Tween 20 (TBST) containing 5% BSA was used as a blocking solution. The membrane was immunoblotted overnight at 4°C with primary antibodies: TLR9 (Novus Biologicals, Littleton), total p65, phosphor p65, NOD2, and CD16 (Cell Signaling Technology, Danvers) at a dilution of 1:1,000 in TBST. Horseradish peroxidase-conjugated anti-rabbit antibody (Cell Signaling Technology, Danvers) was used for secondary labeling at 1:1,000 in TBST for 1 hour at room temperature. The membrane was reprobed with GAPDH rabbit polyclonal antibody (Proteintech, Rosemont) at 1:5,000 in TBST. A horseradish peroxidase-conjugated anti-rabbit (Cell Signaling Technology, Danvers) was used for secondary labeling at 1:5,000 in TBST for 1 hour at room temperature. Protein bands were visualized by enhanced chemiluminescence (ECL, GE Healthcare, Chicago).

### AAT treatment

Lyophilized AAT (Prolastin-C) was reconstituted with deionized water, following the manufacturer’s instruction, and the reconstituted AAT was stored at -80°C. To examine whether AAT is able to mitigate the effect of α-defensins on inducing the expression of inflammatory cytokines, CXCL8, IL-1b, and TNFα, MDMs were incubated with α-defensins only or α-defensins and AAT together for 18 hours. The cells were lysed for RNA extraction using the Qiagen RNeasy kit (Qiagen, Hilden), and the expression levels of the cytokines were compared between MDM controls and α-defensin-treated MDMs using qRT-PCR.

### Statistical analysis

Results are expressed as mean and standard deviation or percentage as appropriate. Comparisons between groups were made by using non-parametric Wilcoxon matched-pairs signed-rank test, non-parametric Mann-Whitney test or one-way analysis of variance (ANOVA). A p-value <0.05 was considered significant. All analyses were performed using the GraphPad Prism 9.3.0 (GraphPad software, San Diego) software package.

## Results

### The level of α-defensins in AATD individuals

**Figure 1:**
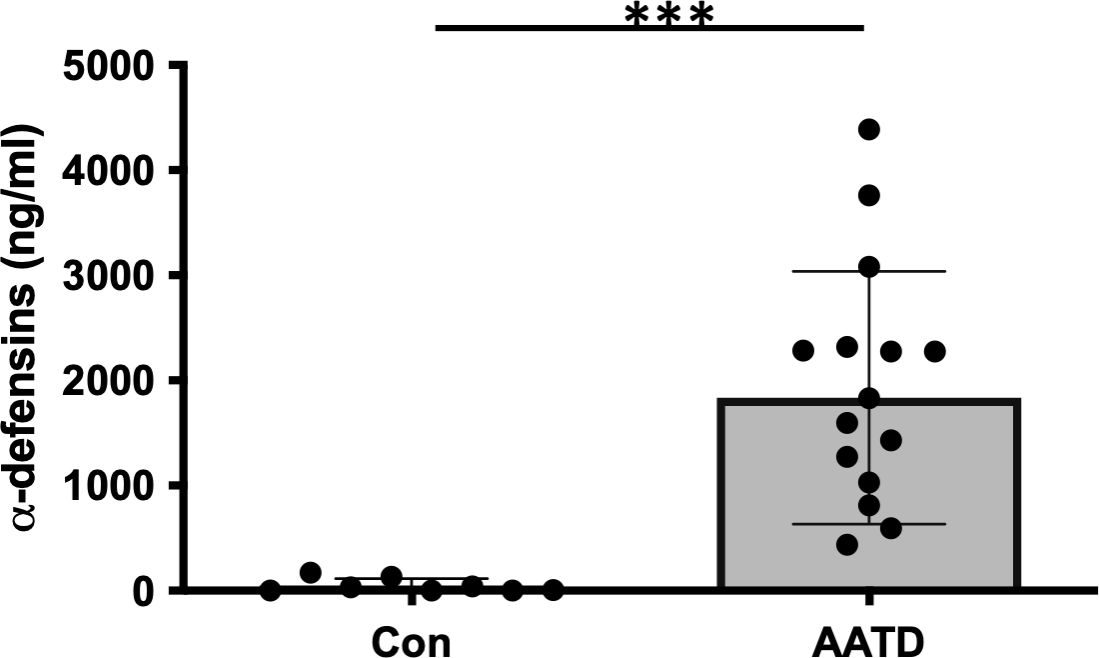
The concentrations of α-defensins increased in AATD individuals. Total BAL fluids were obtained from control and AATD individuals. The concentration of α-defensins was measured using ELISA and compared between the two groups. Statistical analysis was conducted using a non-parametric Mann-Whitney test. Statistical significance is denoted by (***) (p-value < 0.001).

BAL fluids were obtained from control and AATD individuals, and the characteristics of the individuals are shown in **Table 1**. The concentration of α-defensins was measured in the BAL fluid samples. We found significantly higher α-defensin levels in the BAL fluid of AATD individuals with Pi*ZZ phenotype (1,836ng/mL +/- 1202.6), compared to 48ng/ml +/- 66.6 in control individuals (**Figure 1**, p-value = 0.0002).

### Phagocytosis and processing of NTHi by α-defensin-exposed MDMs

**Figure 2:**
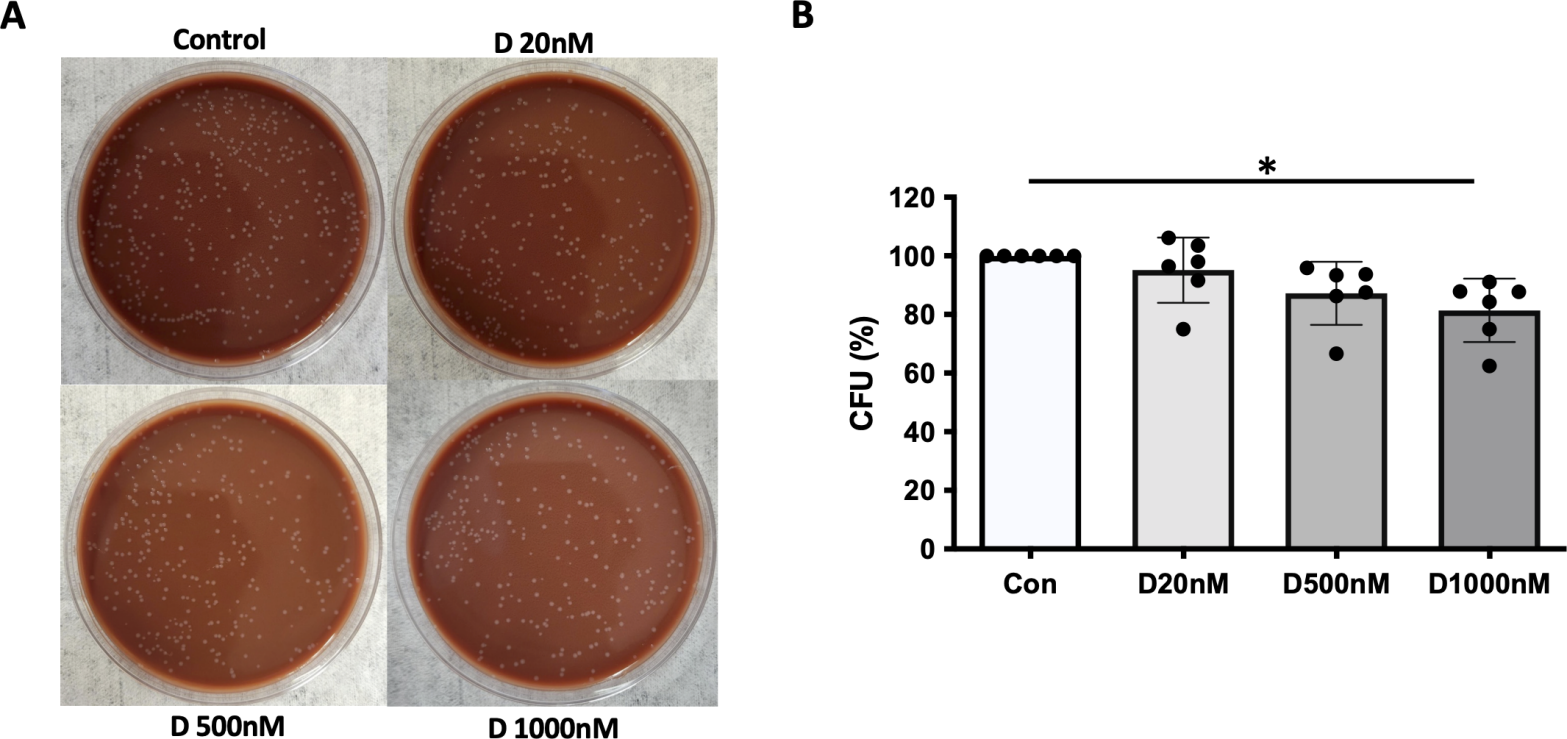
NTHi phagocytosis by MDM inhibited by α-defensins. MDMs were incubated with three different concentrations of α-defensins, 20, 500, and 1000nM overnight and infected with NTHi for three hours. **(A)** MDMs were lysed, and a dilution of cell lysate was cultured on chocolate agar plate overnight. **(B)** The number of bacterial colonies, colony forming units (CFU), was counted per sample. CFU from MDM control was set to 100% and the number of the other samples were normalized to control. Statistical analysis was conducted using one-way ANOVA. Statistical significance is denoted by (*) (p-value < 0.05).

**Figure 3:**
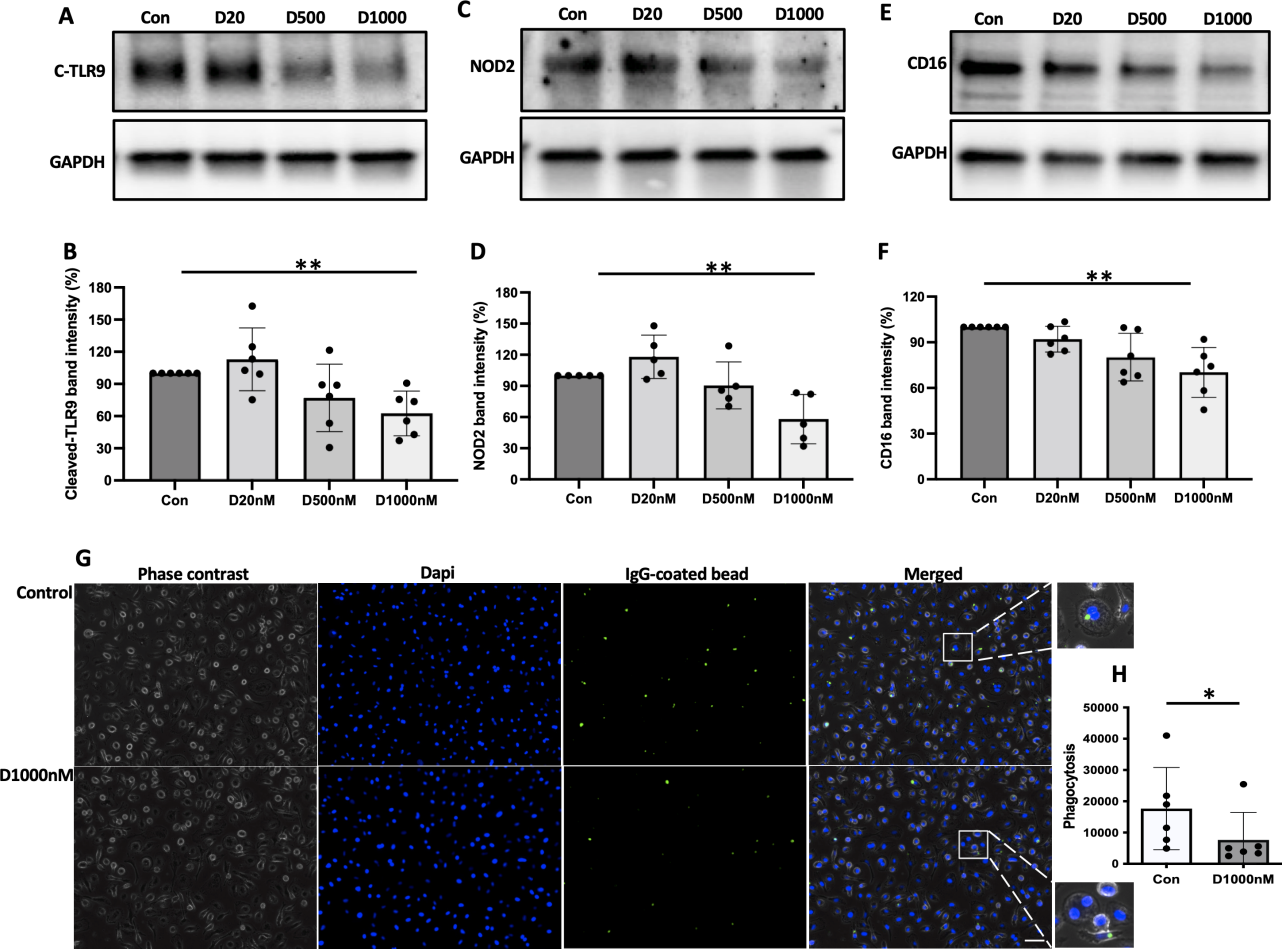
The level of pattern recognition molecules reduced by α-defensins. Total proteins were isolated from MDM controls and α-defensin-treated MDMs at three hours of NTHi infection and subjected to SDS-PAGE to examine **(A)** the cleaved form of TLR9, **(C)** NOD2, and **(E)** CD16 in the cells. Note that cleaved form of TLR9 is an indicator for the TLR9 activation. NOD2 and CD16 are pattern-recognizing molecules. **(B, D, F)** Band intensities of the molecules were quantified using NIH ImageJ and compared among the NTHi-infected MDMs. Statistical analysis was conducted using one-way ANOVA. Statistical significance is denoted by (**) (p-value < 0.01). **(G)** MDMs were incubated with 1000nM of α-defensins overnight. **(H)** MDMs control and the α-defensin-treated MDMs were incubated with IgG-coating latex beads (Green). MDMs phagocytosing the latex beads are visualized using a fluorescence microscope; bar 30 μm. Green fluorescent intensity was normalized to the number of MDMs per sample and compared between MDM controls and α-defensin-treated MDMs. More than 1,000 cells, originating from six separate experiments, were evaluated for each MDM group. Statistical analysis was conducted using Wilcoxon test. Statistical significance is denoted by (*) (p-value < 0.05).

We previously reported that α-defensins suppress the phagocytic ability of MDMs by inhibiting cell motility and macrophages differentiation. Using an antibiotic protection assay, we compared the number of internalized NTHi between control- and α-defensin-treated MDMs. A high concentration of α-defensins decreased the number of internalized NTHi by the MDMs in a concentration-dependent manner, indicating that α-defensins suppress the bacterial up-take (**Figures 2A, B**, p-value = 0.0097). These results are consistent with our previous findings on the effect of α-defensins on phagocytosis (20). Post-engulfment NTHi processing by MDM is triggered by recognition of bacterial DNA via specific intracellular pattern recognition molecules. We investigated two intracellular pattern recognition pathways, TLR9 and NOD2 because of their previous reported role in the recognition and phagocytosis of NTHi by host cells (30–32). We measured lower level of cleaved TLR9 in MDMs treated with a high concentration of α-defensins (**Figures 3A, B**, p-value = 0.004). Similarly, the level of NOD2 was significantly decreased by α-defensins (**Figures 3C, D**, p-value = 0.003). Transcriptionally, α-defensin did not change the expression of *NOD2* and *TLR9* in α-defensin-treated MDMs, compared to MDM controls (**Supplementary Figures 1A, B**). All these results support that α-defensins reduce the ability of MDMs to internalize and process NTHi.

### Phagocytosis of IgG coated beads by α-defensin-exposed MDMs

It was previously reported that α-defensins inhibit the activity of NK cells by reducing the level of CD16 (33). CD16, also known as FcγRIII, is a low-affinity receptor for immunoglobulin G (IgG) and plays an important role in the phagocytosis of antibody-coated targets. IgG-mediated phagocytosis against microbial agents or antigens is essential to control bacterial infection in the lower respiratory tract (34). We compared the protein level of CD16 between control and α-defensin-exposed MDMs at three hours after NTHi infection. The result shows that the CD16 protein level was significantly reduced by α-defensins in NTHi-infected MDMs (**Figures 3E, F**, p-value = 0.004). The reduced level of CD16 could impair macrophage phagocytosis of complement-opsonized and antibody-coated targets and inhibit bacteria killing *in vivo* (35, 36). To examine whether α-defensins inhibit IgG-mediated phagocytosis, we performed a phagocytosis assay using fluorescently labeled IgG-coating latex beads and compared the phagocytosis rate between control- and α-defensin-exposed MDMs. The result shows that the phagocytosis rate of IgG-coated latex beads was significantly reduced by α-defensins, as shown in **Figures 3G, H** (p-value = 0.031).

### NTHi adherence to α-defensin-exposed MDMs

**Figure 4:**
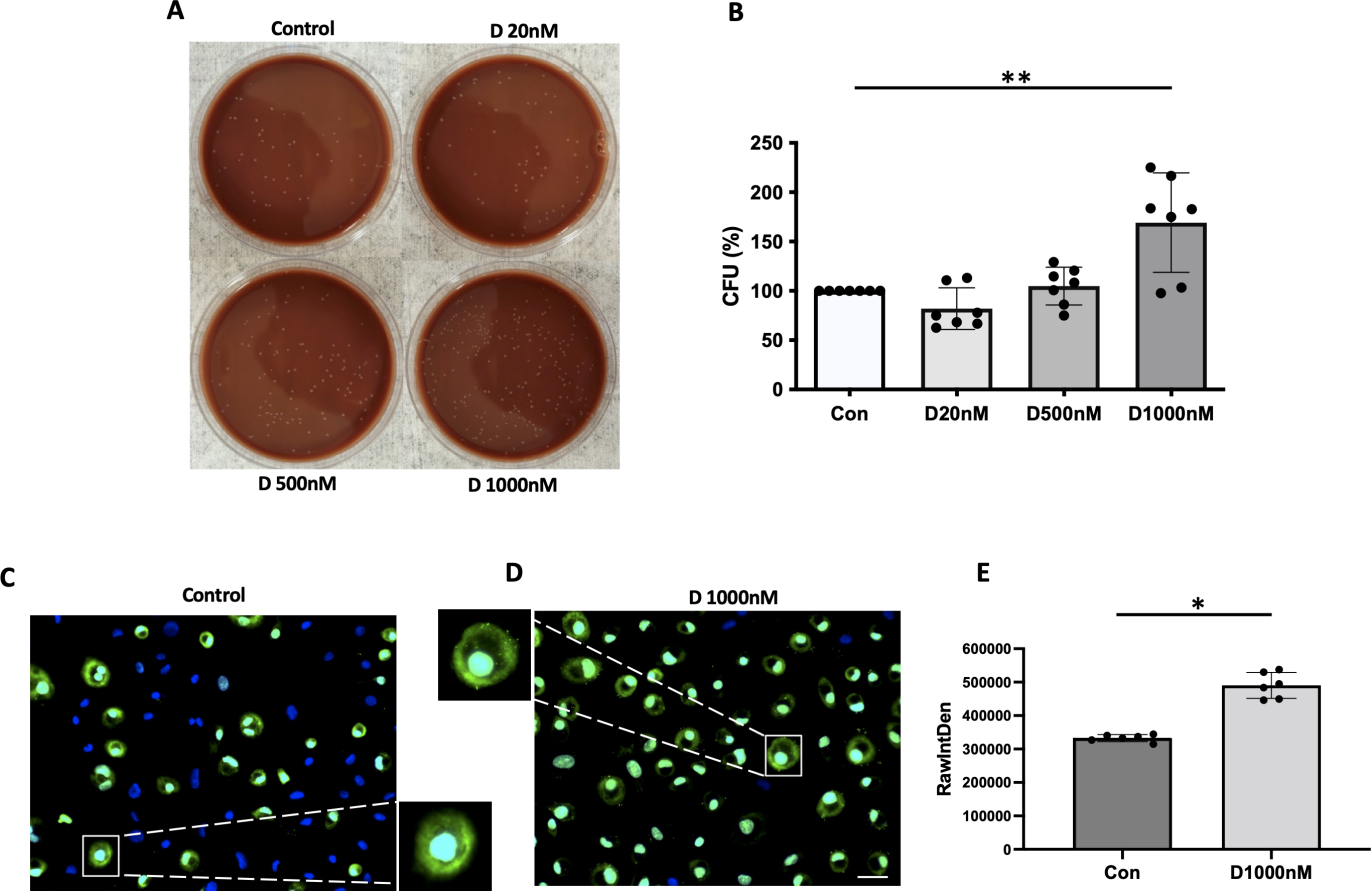
The binding ability of NTHi to MDM enhanced by α-defensins. MDMs were incubated with three different concentrations of α-defensins, 20, 500, and 1000nM overnight and infected with NTHi for three hours. Unbound NTHi were washed with PBS. **(A)** MDMs were lysed, and a dilution of cell lysate was cultured on chocolate agar plate overnight. **(B)** The number of bacterial colonies was counted per sample and compared among the samples. The bacterial colony number of MDM control was set to 100%, and the number of the other samples were normalized to MDM control. Statistical analysis was conducted using one-way ANOVA. Statistical significance is denoted by (**) (p-value < 0.01). MDMs were incubated in the absence or presence of α-defensins overnight and incubated with NTHi for three hours. Unbound NTHi were washed with macrophage differentiation media without antibiotics, and extracellular bacteria were killed by gentamicin. **(C)** MDM controls and **(D)** α-defensin-treated MDMs were incubated with SYTOX Green Nucleic Acid Stain, which binds to genomic DNAs. Bacterial genomic DNAs on the plasma membrane of MDMs are visualized using a fluorescence microscope; bar 30 μm. **(E)** Green fluorescent intensity excluding nucleus was normalized to the number of MDMs per sample and compared between MDM controls and α-defensin-treated MDMs. ~300 cells, originating from six separate experiments, were evaluated for each MDM group. Statistical analysis was conducted using Wilcoxon test. Statistical significance is denoted by (*) (p-value < 0.05).

α-defensins are known to increase the adherent ability of NTHi to lung epithelial cells. We found that a high concentration of α-defensins promote NTHi binding to MDMs. We incubated α-defensin-exposed MDMs with NTHi for three hours and removed unbound bacteria. After overnight culture, serial dilution of α-defensin-exposed MDM lysates showed higher % CFU than MDM controls, suggesting that the number of cell-associated bacteria, including both internalized bacteria and adherent bacteria to MDMs, was significantly increased by α-defensins (**Figures 4A, B**, p-value = 0.004). To specifically visualize the cell-associated bacteria, we used SYTOX Green Nucleic Acid Stain (**Figures 4C, D**). Green-fluorescence-labeled NTHi were detected primarily on the membrane of α-defensin-treated MDMs vs MDMs. Quantification of the green signal shows that the number of cell-associated bacteria is significantly higher in α-defensin-exposed MDMs than MDM controls (**Figure 4E**, p-value = 0.0312).

### Expression and secretion of inflammatory cytokines by α-defensin-exposed MDMs

**Figure 5:**
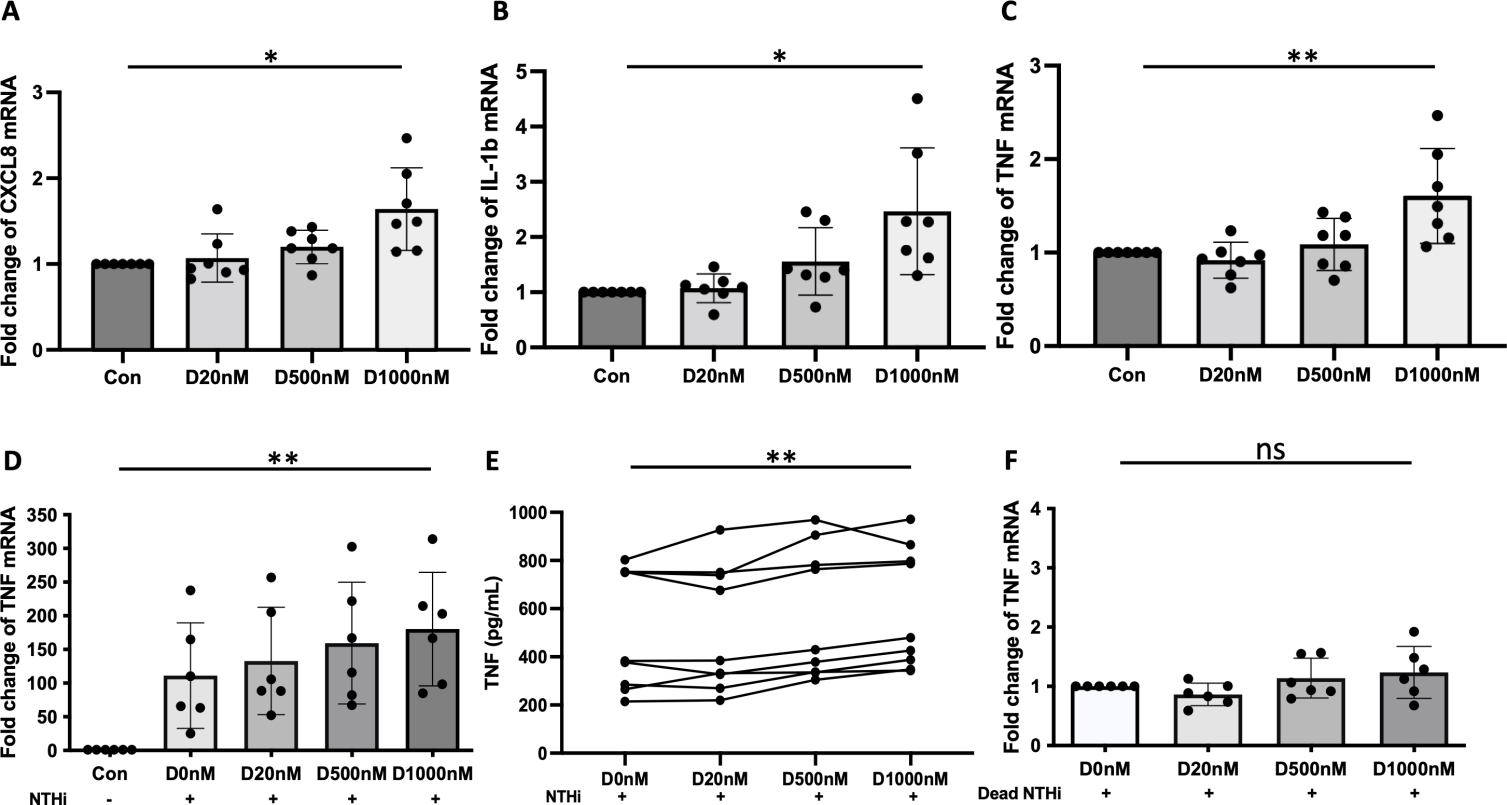
The effect of α-defensins on the expression of inflammatory cytokines in MDMs. MDMs were incubated with three different concentrations of α-defensins, 20, 500, and 1000nM, overnight. **(A–C)** The expression levels of inflammatory cytokines, CXCL8, IL-1β, and TNFα, were compared between MDM controls and α-defensin-treated MDMs. Their relative expression is represented by fold change. **(D)** MDM controls and α-defensin-treated MDMs were infected with NTHi for three hours, and the expression levels of TNFα were compared between them. **(E)** The conditioned media were collected from the NTHi-infected cells, and the concentration of TNFα was measured in the media using ELISA assay. **(F)** MDM controls and α-defensin-treated MDMs were infected with heat-killed NTHi for three hours, and the expression levels of TNFα were compared between them. Statistical analysis was conducted using one-way ANOVA. Statistical significance is denoted by (*) (p-value < 0.05) and (**) (p-value < 0.01).

Excessive amounts of α-defensins induce the expression of inflammatory cytokines in lung epithelial cells (6, 7). We incubated MDMs with increasing concentrations of α-defensins and compared the gene expression of pro-inflammatory cytokines between control- and α-defensin-treated MDMs. The result showed that α-defensins induce the expression of *CXCL8*, *IL-1β*, and *TNFα* in a concentration-dependent manner (**Figure 5A**, p-value = 0.019, 5B, p-value = 0.014, 5C, p-value = 0.006). The levels of endotoxin were undetectable in the α-defensin preparate used in this assay, indicating that α-defensins themselves indeed induced the expression of inflammatory cytokines in MDMs (data not showed). To examine whether α-defensins could exacerbate bacteria-mediated inflammation, we compared the expression levels of inflammatory cytokines in NTHi-infected MDMs in the absence and presence of α-defensins. Only the expression of *TNFα* was significantly increased by a high concentration of α-defensins in NTHi-infected MDMs (**Figure 5D**, p-value = 0.003), while expression of *CXCL8* and *IL-1β* showed no significant differences between NTHi-infected MDMs incubated with and without α-defensins (**Supplementary Figures 2A, B**). Moreover, secreted TNFα measured by ELISA, was significantly increased by a high concentration of α-defensins in NTHi-infected cells (**Figure 5E**, p-value = 0.0007).

To examine whether the viability of NTHi is required for the effect of α-defensins, we conducted a similar experiment using heat-killed NTHi. Interestingly, a high concentration of α-defensins did not increase the expression of *TNFα* in heat-killed bacteria-infected MDMs (**Figure 5F**).

**Figure 6:**
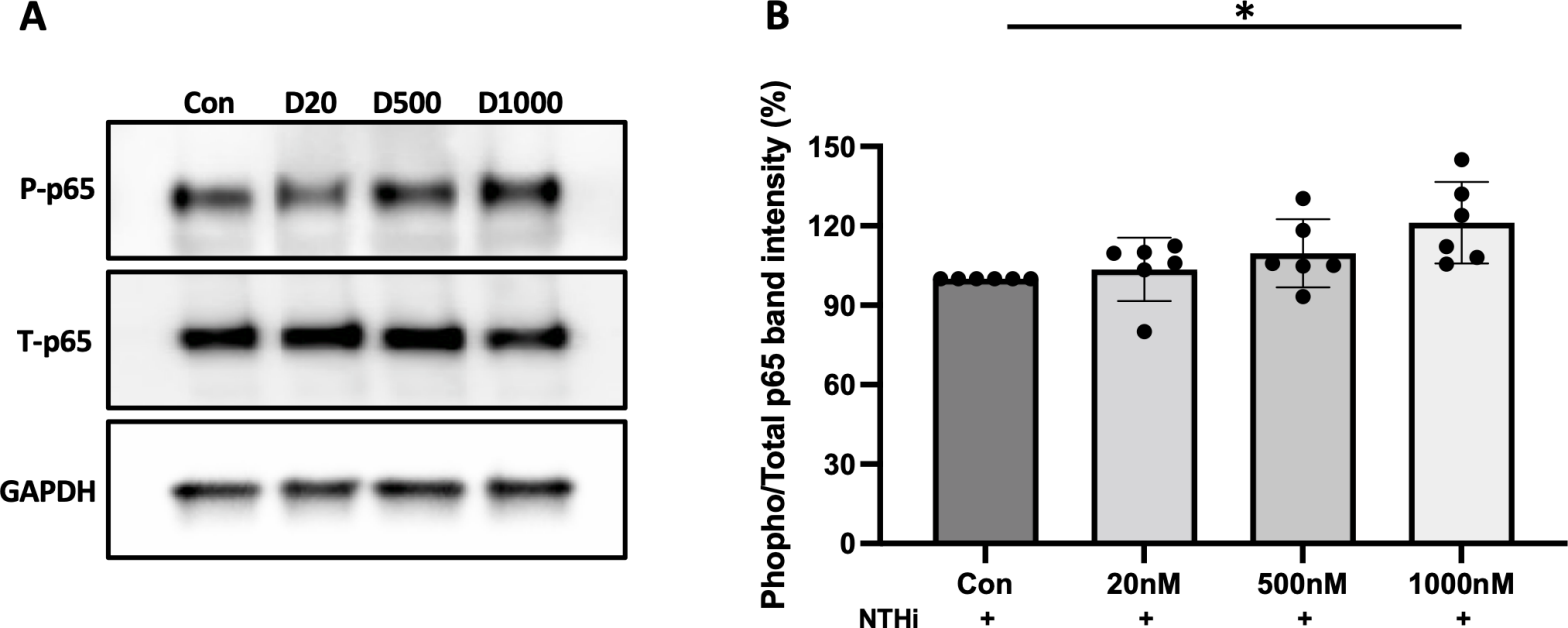
The phosphorylation of p65 increased by α-defensins. Total proteins were isolated from MDMs control and α-defensin-treated MDMs at three hours of NTHi infection and subjected to SDS-PAGE to examine the phosphorylation of p65, an indicator for the activation of NF-κB signaling, in the cells. **(A)** The levels of total p65 and phosphorylated p65 were analyzed via SDS-PAGE. **(B)** The protein bands were quantified using NIH ImageJ software and a ratio of phosphorylated p65 to total p65 was measured and compared between MDM controls and α-defensin-treated MDMs. Statistical analysis was conducted using one-way ANOVA. Statistical significance is denoted by (*) (p-value < 0.05).

Next we asked whether TNFα expression in NTHi and α-defensin-treated cells is under NF-κB control, as NF-κB is the master transcription factor which regulates the expression of TNFα (37), and where phosphorylation of p65 indicates the activation of NF-κB signaling (38). We demonstrate that the level of the phosphorylated p65 to total p65 was increased as the concentration of α-defensins was increased, indicating that the activation of NF-κB by α-defensins could be responsible for the increased *TNFα* expression in α-defensin-treated and NTHi-infected cells (**Figures 6A, B**, p-value = 0.027). Our result shows that a high concentration of α-defensins enhances the expression of *TNFα* in NTHi-infected MDMs via NF-κB signaling activation. Moreover, the α-defensin-NTHi additive effect on MDMs is dependent on the viability of NTHi, suggesting that MDMs primed by α-defensins may respond faster and augmented TNFα secretion during NTHi infection.

### TLR4-mediated expression of TNFα in NTHi-infected and α-defensin-exposed MDMs

**Figure 7:**
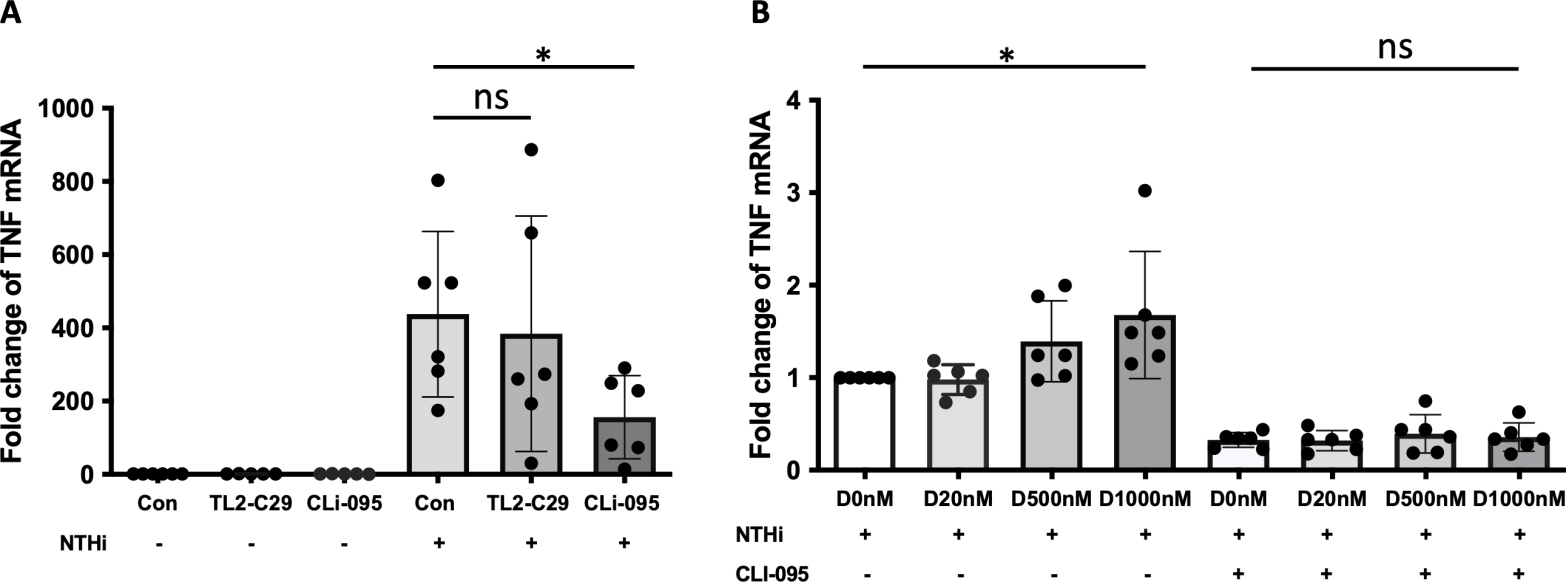
α-defensin-inducing TNFα expression reduced by TLR4 inhibitor. **(A)** MDMs were pre-treated with TL2-C29 or CLi-095 for one hour and then incubated with NTHi for three hours. Total RNAs were isolated from the samples and the expression levels of TNFα were compared among the samples. *Denotes statistical significance (p < 0.05) according to Wilcoxon test. **(B)** The activation of TLR4 was inhibited by CLi-095 in MDM control and α-defensin-treated MDMs, and the cells were incubated with NTHi for three hours. The expression levels of TNFα were compared among the samples. Statistical analysis was conducted using one-way ANOVA. Statistical significance is denoted by (*) (p-value < 0.05), and (ns) indicates no statistical difference among the samples.

It was reported that NTHi activates NF-κB via TLR2 (39), and NTHi initiates immune response by activating TLR4/NF-κB signaling (40). To delineate between TLR2 vs. TLR4 signaling, we used the TLR2 and TLR4 pharmacological inhibitors TL2-C29 (41) and CLI-095 (42), respectively. At baseline, the expression levels of *TLR2* and *TLR4* were similar in control and α-defensin-treated MDMs (**Supplementary Figure 3A**). Moreover, in the absence of NTHi infection, neither TLR2 nor TLR4 inhibitors had an effect on *TNFα* expression in MDMs. After NTHi infection, the *TNFα* expression was similar in TL2-C29-treated as in MDM controls, indicating that TLR2 does not mediate the expression of *TNFα* in NTHi-infected MDMs. However, CLI-095, a TLR4 inhibitor, significantly decreased the *TNFα* expression in NTHi-infected and α-defensin-exposed MDMs vs. NTHi-infected MDM controls. This indicates that TLR4 mediates the NTHi-induced expression of *TNFα* in MDMs (**Figure 7A**, p-value = 0.011).

To examine whether TLR4 is necessary in α-defensin-mediated *TNF* expression in NTHi-infected MDMs, we incubated α-defensin-treated MDMs with CLI-095 and then infected the MDMs with NTHi. We noticed lower *TNFα* expression level in CLI-095 treated, α-defensin exposed and NTHi infected MDMs compared to α-defensin exposed and NTHi infected MDMs (**Figure 7B**, p-value = 0.036). It indicates that TLR4 signaling increases α-defensin-mediated *TNFα* expression in NTHi-infected MDMs. The expression level of *TLR4* was gradually increased by α-defensins in MDMs, but the increase was not statistically significant (**Supplementary Figure 3B**). However, the expression level of *TLR4* was significantly increased by α-defensins in NTHi-infected cells (**Supplementary Figure 3C**, p-value = 0.048). The protein level of TLR4 was also significantly higher in α-defensin-treated MDMs than MDM controls (**Supplementary Figures 3D, E**, p-value = 0.0312).

### Exogenous AAT administration effect on the expression of inflammatory cytokines in α-defensin-exposed MDMs

**Figure 8:**
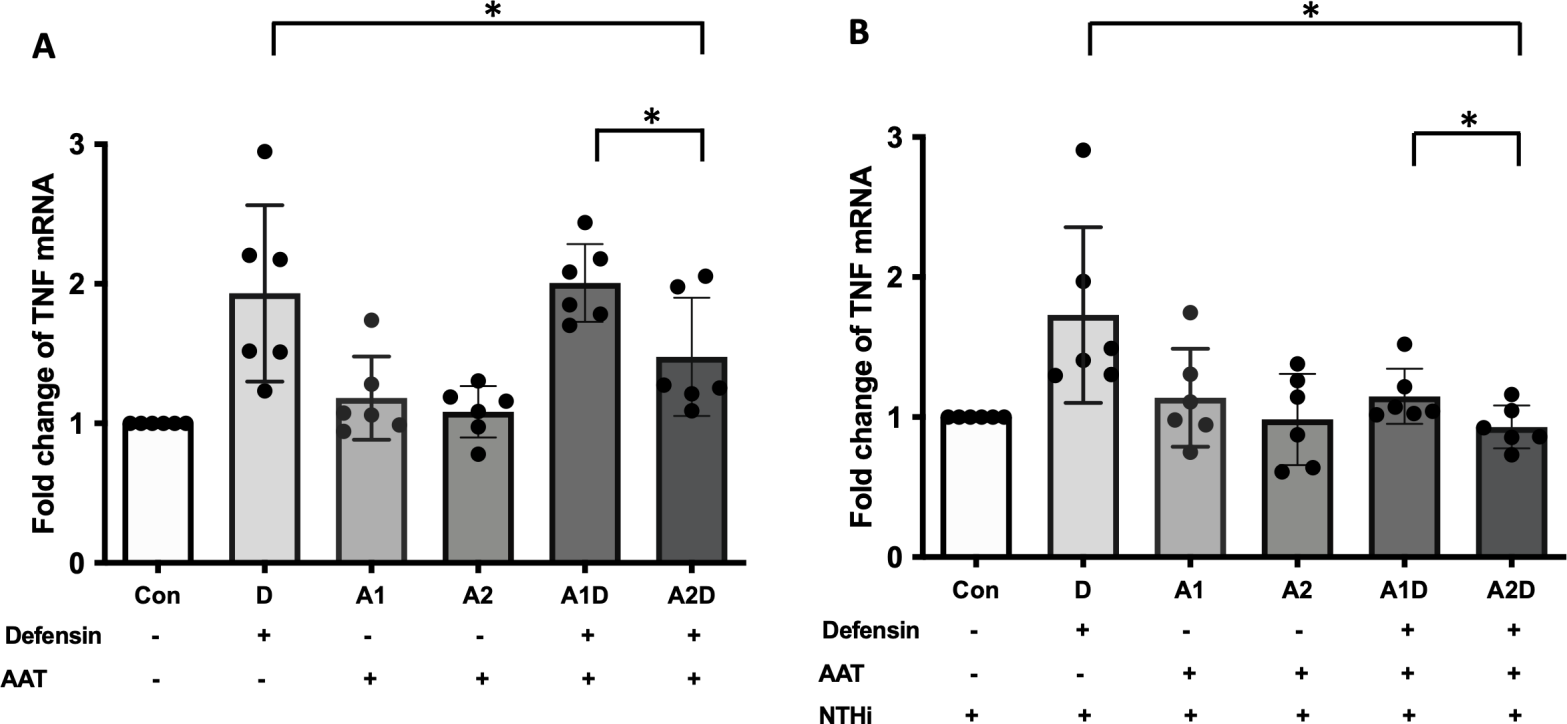
The effect of α-defensins on the expression of TNFα blocked by AAT. MDMs were incubated with α-defensins only or α-defensins and AAT together. **(A)** To examine whether AAT could block the effect of α-defensins on inducing the expression of TNFα, the expression levels of TNFα were compared among six different samples, control, 1 μM of α-defensin-treated MDMs, 1 μM of AAT-treated MDMs, 4 μM of AAT-treated MDMs, 1 μM of α-defensin- and 1 μM of AAT-treated MDMs, and 1 μM of α-defensin- and 4 μM of AAT-treated MDMs. **(B)** The six different samples were infected with NTHi for three hours. Total RNAs were isolated from the NTHi-infected MDMs, and the expression levels of TNFα were compared among the samples. *Denotes statistical significance (p < 0.05) according to Wilcoxon test.

AAT can bind to α-defensin and inactivate its downstream signaling (24, 43). Therefore, it was intriguing to examine whether exogenous AAT treatment can reduce the pro-inflammatory cytokine secretion increased by α-defensins in MDMs. MDMs were incubated with 1μM of α-defensins and low, 1μM or high, 4μM of AAT. The higher concentration AAT significantly reduced the expression of *TNFα* in the MDMs (p-value = 0.0312), while the lower concentration of AAT failed to reduce α-defensin-induced *TNFα* expression (**Figure 8A**). The expression levels of *CXCL8* and *IL-1β* were not changed by AAT treatment in α-defensin-exposed MDMs (data not shown). Moreover, AAT treatment was able to reduce the expression of *TNFα* in α-defensin-treated and NTHi-infected MDMs both at 1μM and 4μM concentration with significantly lower TNFα expression level in 4μM AAT-treated MDMs vs. 1μM AAT-treated cells (**Figure 8B**, p-value = 0.0312).

### Phagocytosis of IgG coated beads by primary human alveolar macrophages in AATD individuals

**Figure 9:**
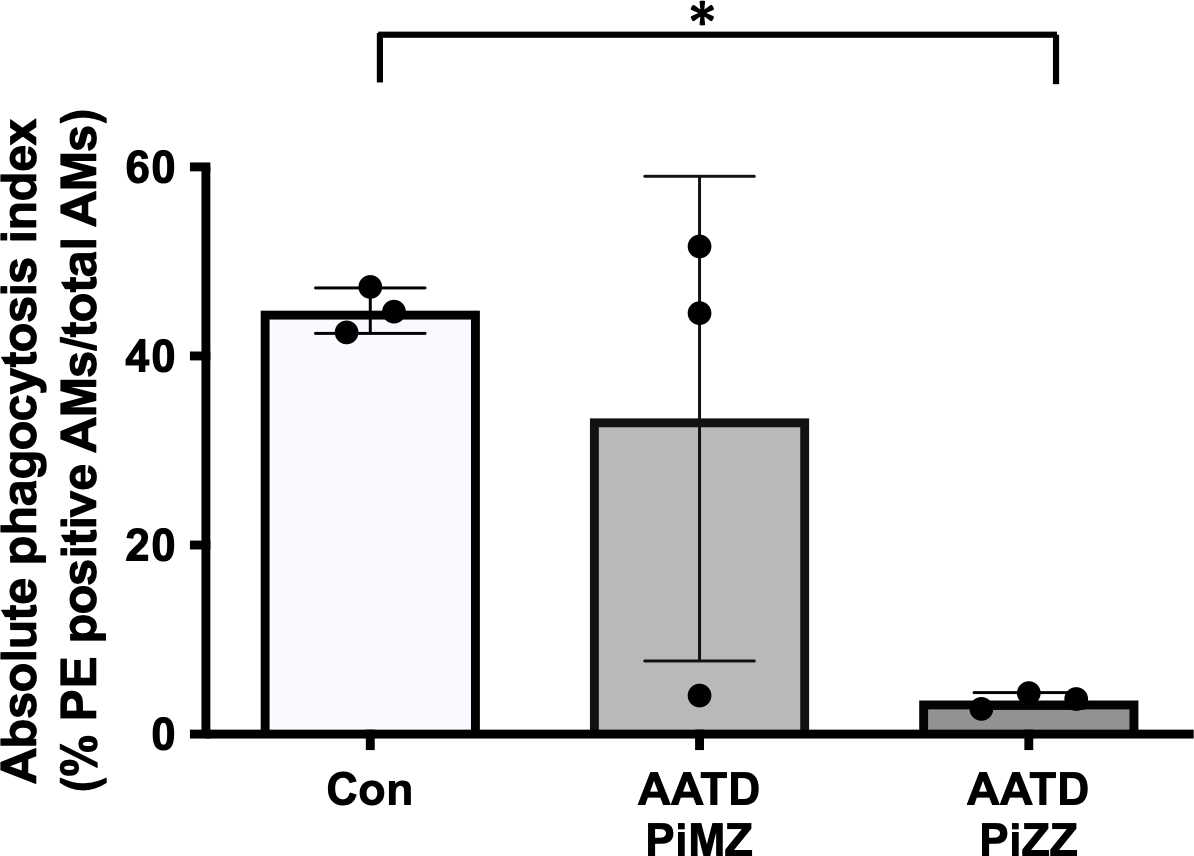
Phagocytosis of IgG-coated bead by primary alveolar macrophages. Primary alveolar macrophages were obtained from the bronchoalveolar lavage fluid of controls and AATD individuals. AATD individuals were divided into two groups of Pi*MZ and Pi*ZZ based on their AAT genotype. The macrophages were incubated with IgG-coated beads for 1 hour at 37°C at 1:5 ratio (AM:beads). Absolute phagocytosis index representing % of primary human AM that engulfed FITC-labeled Fc-coated targets. Note lower phagocytosis index in Pi*ZZ AM vs. Pi*MZ and healthy, non-smokers AM. * Denotes statistical significance (p<0.05) according to one-way ANOVA, Tukey’s multiple comparison test.

Lastly, primary human alveolar macrophages were isolated from control and AATD individuals (patient characteristics of the individuals are shown in **Table 2**). The phagocytosis rate was significantly reduced in AATD with Pi*ZZ genotype, compared to the control (**Figure 9**, p-value = 0.0336), when alveolar macrophages were co-incubated with IgG-coated fluorescent beads (AM:beads ratio 1:5, 1h).

**Table 2:** Characteristics of controls and AATD individuals used for the phagocytosis assay of primary alveolar macrophages.

Characteristic	PiMM (n=3)	PiMZ (n=3)	PiZZ (n=3)
Age	57.3 ± 19.8	55 ± 8.2	39.3 ± 4.6
Gender (M/F)	1/2	0/3	0/3
FEV1% predicted	N/A	105 ± 7.1	89.6 ± 18.7
Current smoker	N/A	No	No
Neutrophil (%)	0.5 ± 0.34	1.6 ± 0.47	0.66 ± 0.94
Macrophage (%)	96 ± 1.26	91.3 ± 3.85	94 ± 0.81
Lymphocyte (%)	2 ± 2.44	7 ± 3.74	5.3 ± 0.47
Eosinophil (%)	0	0	0

Definition of abbreviations: PiMM, individuals homozygous for normal PiM allele; PiMZ, individuals heterozygous for normal PiM and mutant PiZ allele; PiZZ, individuals homozygous for mutant PiZ allele; N/A, not available; FEV1, forced expiratory volume in one second. Data are presented as mean ± standard deviation (SD).

## Discussion

**Figure 10:**
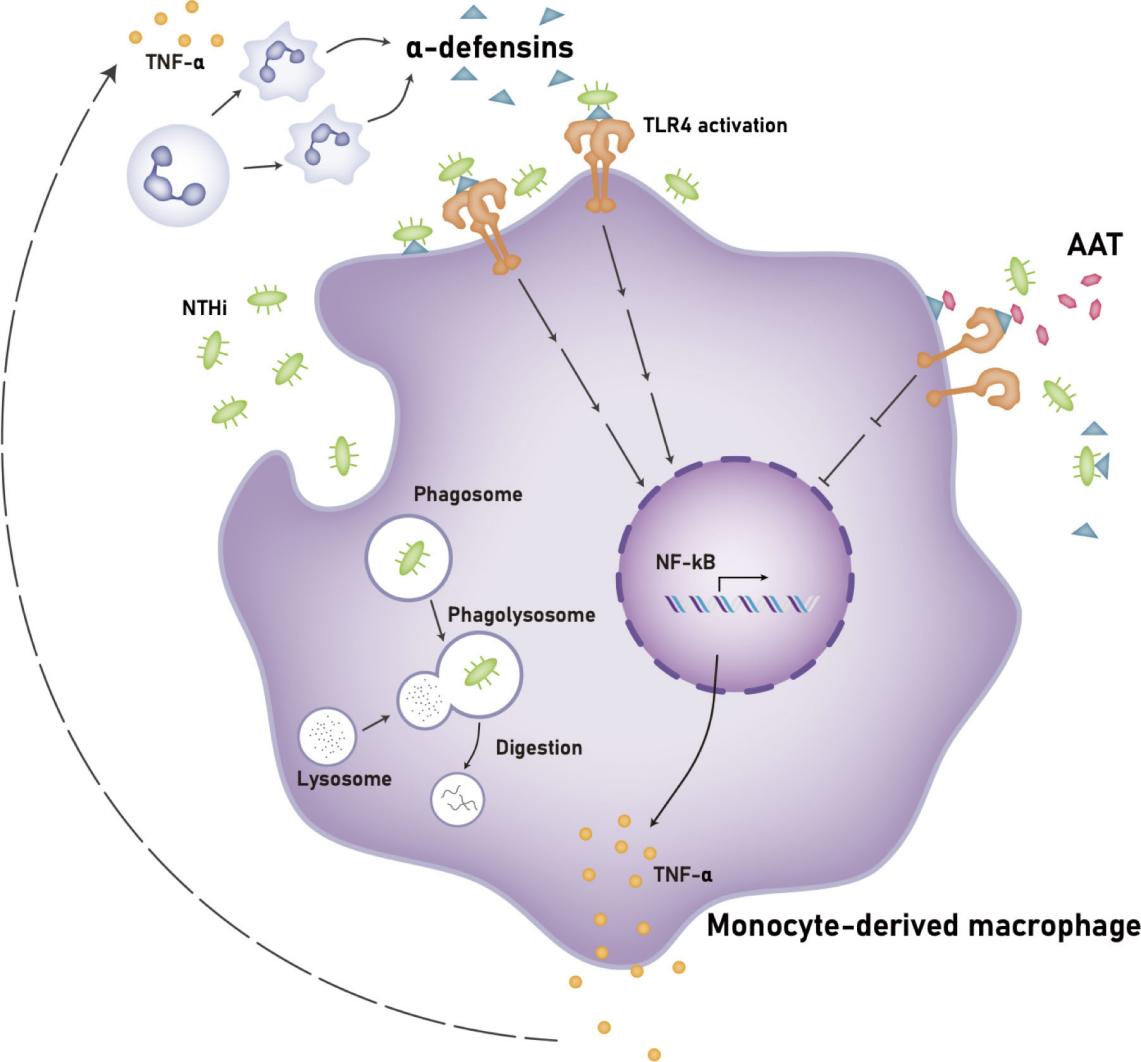
Proposed mechanism of the effects of a high concentration of α-defensins on NTHi-infected macrophages. A high concentration of α-defensins enhances NTHi binding to the macrophage membranes but inhibits NTHi engulfment by MDMs. In the presence of α-defensins, NTHi promotes the activation of TLR4, and subsequently activates NF-κB through the phosphorylation of p65, increasing the expression and secretion of TNFα. The secreted TNFα induces neutrophil apoptosis, leading to a high level of α-defensins. Exogenous AAT at physiologic levels binds to α-defensins to reduce the activation of TLR4 during NTHi infection, leading to reduced TNFα expression and secretion. Definition of abbreviations: NTHi, nontypeable *Haemophilus influenzae*; AAT, alpha1-antitrypsin.

Our study demonstrates that a high concentration of α-defensins causes NTHi binding to, but inhibits NTHi phagocytosis by macrophages. In addition, we found that α-defensins increase the expression of pro-inflammatory cytokines in macrophages and the secretion of TNFα through the activation of TLR4/NF-kB p65 signaling in the NTHi-infected macrophages. Moreover, we show for the first time that AAT supplementation and TLR4 inhibitors ameliorate macrophages pro-inflammatory cytokine expression and secretion. Our findings represent a novel mechanisms by which excessive amounts of α-defensins could exacerbate NTHi colonization in the AATD lung and possible therapeutic interventions that may contract the effect of high concentrations of α-defensins in AATD airways (**Figure 10**).

Many lung diseases including AATD are characterized by a high number of alveolar neutrophils, a high concentration of α-defensins, and persistent bacterial infection (1, 44, 45). It is paradoxical because neutrophils are professional phagocytes which kill and clear invading pathogens in the lung, and α-defensins have antibacterial activities. These two mechanisms are supposed to clear bacterial infection, but various respiratory pathogens including *Pseudomonas aeruginosa, Haemophilus influenzae, Moraxella catarrhalis, Streptococcus pneumoniae* are persistent in patients with chronic lung inflammation, like CF, COPD, and AATD (46, 47). Although it has been generally accepted that α-defensins are only beneficial to host cells, our current and published data suggest that a high concentration of α-defensins could cause and exacerbate lung tissue injuries by inducing the expression of inflammatory cytokines by airway macrophages, impairing cell membrane permeability and inhibiting macrophage phagocytosis (20, 48–50).

NTHi infection is recurrent in patients with COPD and AATD (51, 52). Similar to COPD patients, where the concentration of α-defensins was higher in BAL fluid of patients with severe obstruction than in mild to moderate COPD patients (53), we show that the concentration of α-defensins is significantly higher in BAL fluid of AATD individuals than control individuals. Although neutrophils are the first responder to microbial infection, the apoptotic neutrophils could lead to a high concentration of α-defensins at the site of infection. This is associated with a lower ability of alveolar macrophages to concomitantly clear up apoptotic neutrophils and bacteria-like targets (28). We indeed found that the ability of alveolar macrophages to engulf IgG-coated beads is impaired in AATD individuals. Moreover, a high concentration of α-defensins is known to enhance bacterial binding to lung epithelial cells. This study found that the phagocytosis of NTHi by MDMs is inhibited by α-defensins, with 1000 nM of α-defensins significantly reducing the MDM phagocytosis of NTHi. As suggested by previous studies, α-defensins did not directly kill NTHi in MDM cultures, because the antimicrobial activity of α-defensins is abolished when the concentration of NaCl is as high as in the cell culture media (14). Also, it has been suggested that the extracellularly released α-defensins are probably not bactericidal *in-vivo* because the concentration of salt in the ELF of patients with chronic lung inflammation is high enough to abolish their bactericidal effect (13, 54). Our novel findings that a high concentration of α-defensins fails to directly kill or phagocytose invading pathogens support the concept that high levels of extracellular α-defensins promote airway colonization and recurrent infections.

As in other chronic diseases characterized by chronic neutrophilic airway inflammation, like cystic fibrosis (15, 55) and diffuse panbronchiolitis (56), we demonstrate that a high concentration of α-defensins, despite their beneficial antibacterial effect (57), exacerbates airway injury by inducing the expression of inflammatory cytokines, CXCL8, IL-1b, and TNFα, not only in epithelial cells, but also in airway macrophages. It was previously reported that the level of CXCL8 is significantly correlated with the concentration of α-defensins in BAL fluid of patients with diffuse panbronchiolitis (56). The higher concentration of CXCL8 is found in the sputum and BAL fluid of patients with COPD, including those with AATD and correlates with the increased neutrophil accumulation (58). In AATD, it is known that uncontrolled production of CXCL8 leads to exaggerated inflammation and lung tissue damage (59, 60). Interestingly, our results show that in NTHi-infected MDMs, a high concentration of α-defensins is pro-inflammatory via TNFα-mediated signaling. The concentration of TNFα is significantly higher in patients with COPD (61), and TNFα is essential in the pathogenesis of lung diseases associated with AATD (62). Indeed, our studies demonstrate that TNFα in NTHi-infected MDMs at the gene expression and protein levels are significantly increased by α-defensins. The mechanism is linked to the ability of α-defensins to attach to macrophages membrane and engage TLR4 downstream signaling via NF-κB/p65 phosphorylation to increase transcription of proinflammatory cytokines, including TNF-α. Moreover, we blocked the TNFα expression when the TLR4 signaling was pharmacologically inhibited in α-defensins-treated and NTHi-infected MDMs, indicating that biological and pharmacological inhibitors targeting TLR4 signaling could alleviate pro-inflammatory effects of α-defensins during AATD infectious exacerbations.

AAT augmentation therapy with weekly intravenous infusion of pooled human serum AAT is the main disease-modifying therapy in individuals with AATD-associated lung disease. AAT supplementation is expected to attenuate α-defensin-causing lung injury because AAT binds to α-defensins (24). Our data confirm that exogenous AAT reduces α-defensin-inducing expression of TNFα in MDMs in the absence and presence of NTHi infection. This is a novel, immunomodulatory effect of AAT molecule, in addition to canonical role of protease inhibition. However, despite AAT inhibition of α-defensins signaling, we know that AATD individuals with frequent exacerbations phenotype continue to have recurrent infections and lung function decline even on augmentation therapy. In these patients, lowering α-defensin levels and signaling via TLR4 inhibition might be of therapeutic importance, to decrease the frequency and severity of AATD exacerbation.

## Data Availability

The original contributions presented in the study are included in the article/**Supplementary Material**. Further inquiries can be directed to the corresponding author.

## Supplementary material

Supplementary Figure 1 The expression levels of NOD2 and TLR9 in α-defensin-treated and NTHi-infected MDMs. MDMs were incubated with three different concentrations of α-defensins, 20, 500, and 1000nM, overnight. MDM controls and α-defensin-treated MDMs were infected with NTHi for three hours, and the expression levels of **(A)** NOD2 and **(B)** TLR9 were compared among the four different samples. Statistical analysis was conducted using one-way ANOVA. (ns) indicates no statistical difference among the samples.

Supplementary Figure 2 The effect of α-defensins on the expression of CXCL8 and IL-1β in NTHi-infected MDMs. MDMs were incubated with three different concentrations of α-defensins, 20, 500, and 1000nM, overnight. MDM controls and α-defensin-treated MDMs were infected with NTHi for three hours, and the expression levels of **(A)** CXCL8 and **(B)** IL-1β were compared among the four different samples. Statistical analysis was conducted using one-way ANOVA. (ns) indicates no statistical difference among the samples.

Supplementary Figure 3 The effect of α-defensins on the expression of TLRs in MDMs. MDMs were incubated with three different concentrations of α-defensins, 20, 500, and 1000nM overnight, and the expression levels of **(A)** TLR2 and (B) TLR4 were compared between MDM controls and α-defensin-treated MDMs. MDM controls and α-defensin-treated MDMs were infected with NTHi for three hours. **(C)** The expression level of TLR4 was compared among the four different samples. Statistical analysis was conducted using one-way ANOVA. Statistical significance is denoted by (*) (p-value < 0.05), and (ns) indicates no statistical difference among the samples. **(D)** The protein level of TLR4 was analyzed via SDS-PAGE. **(E)** The protein bands were quantified using NIH ImageJ software and compared between MDM controls and 1000nM of α-defensin-treated MDMs using a non-parametric Wilcoxon matched-pairs signed-rank test. Statistical significance is denoted by (*) (p-value < 0.05).
