# Supplementary figures and images for: Alpha-defensins increase NTHi binding but not engulfment by the macrophages enhancing airway inflammation in Alpha-1 antitrypsin deficiency
Source: Front Immunol. 2025 Feb 12;16:1543729. doi: 10.3389/fimmu.2025.1543729 (PMC11861504; doi:10.3389/fimmu.2025.1543729)

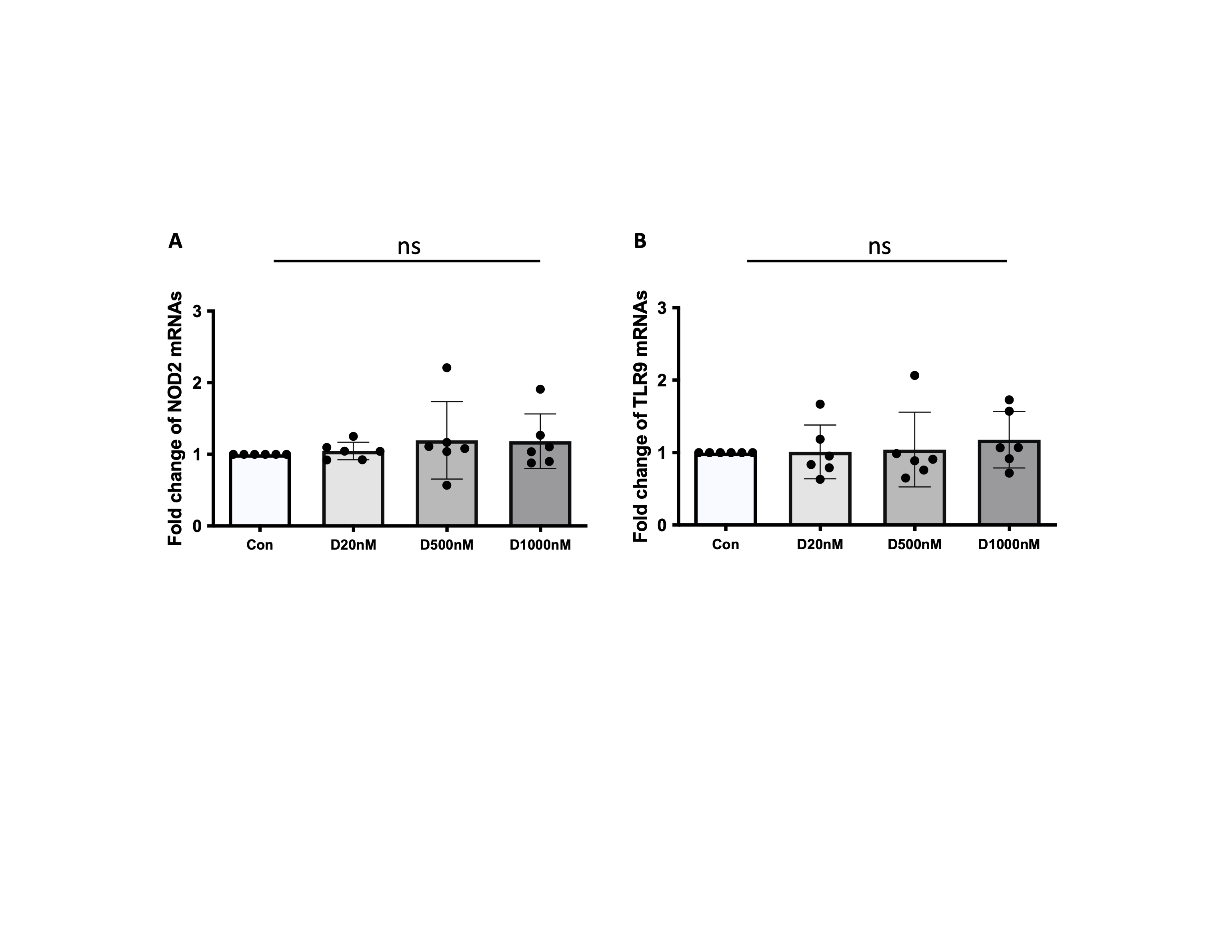

Supplement: Supplementary Figure 1 — The expression levels of NOD2 and TLR9 in α-defensin-treated and NTHi-infected MDMs. MDMs were incubated with three different concentrations of α-defensins, 20, 500, and 1000nM, overnight. MDM controls and α-defensin-treated MDMs were infected with NTHi for three hours, and the expression levels of (A) NOD2 and (B) TLR9 were compared among the four different samples. Statistical analysis was conducted using one-way ANOVA. (ns) indicates no statistical difference among the samples. [file Image1.tiff]

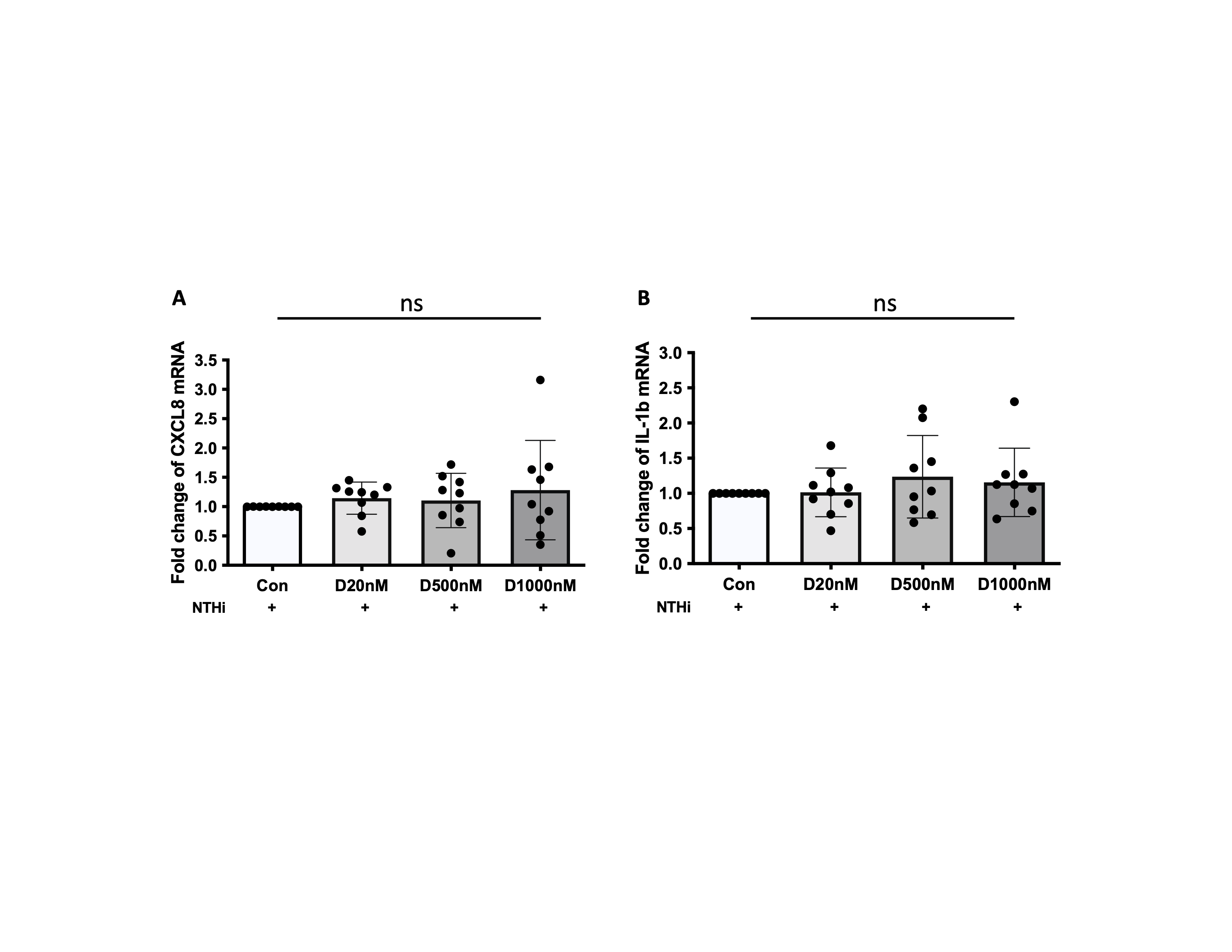

Supplement: Supplementary Figure 2 — The effect of α-defensins on the expression of CXCL8 and IL-1β in NTHi-infected MDMs. MDMs were incubated with three different concentrations of α-defensins, 20, 500, and 1000nM, overnight. MDM controls and α-defensin-treated MDMs were infected with NTHi for three hours, and the expression levels of (A) CXCL8 and (B) IL-1β were compared among the four different samples. Statistical analysis was conducted using one-way ANOVA. (ns) indicates no statistical difference among the samples. [file Image2.tiff]

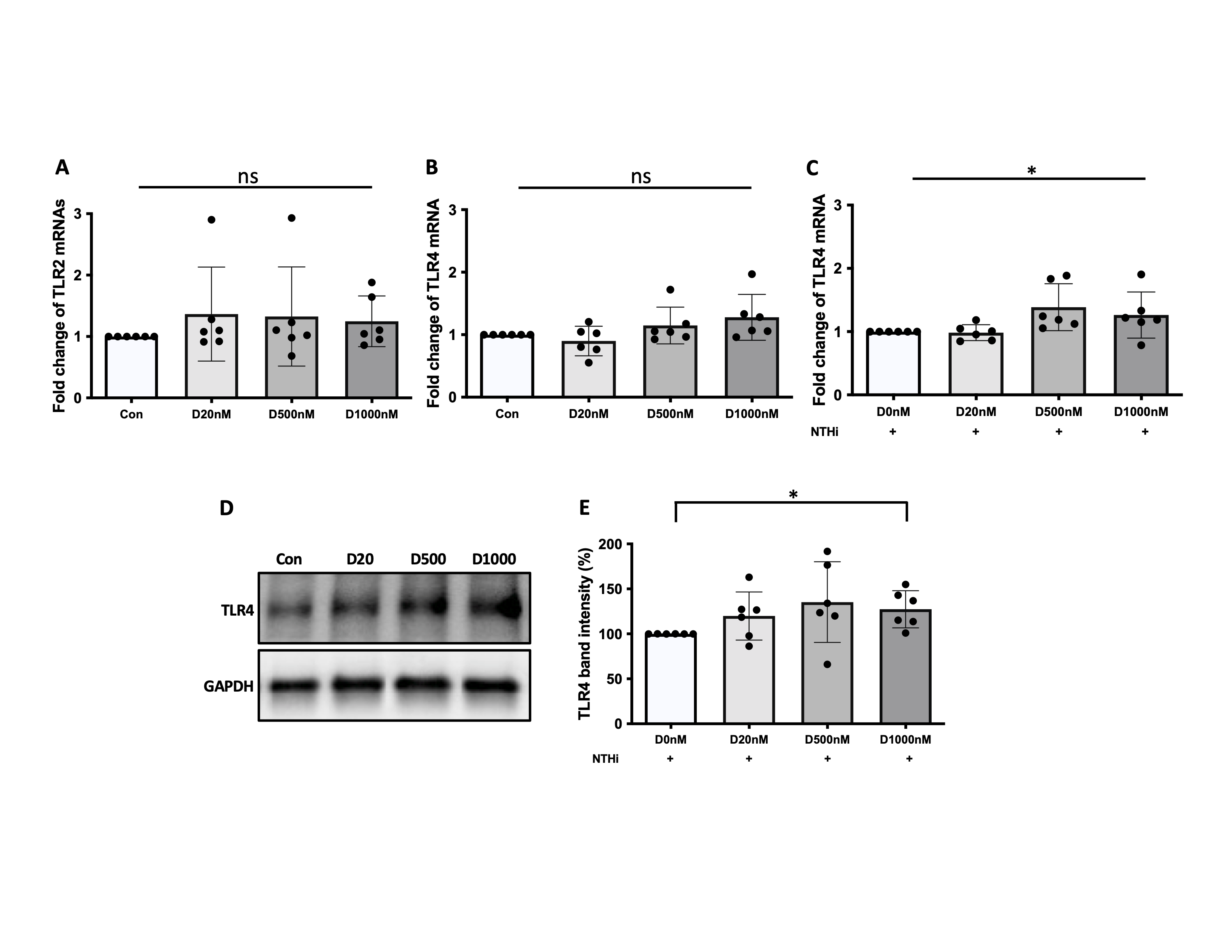

Supplement: Supplementary Figure 3 — The effect of α-defensins on the expression of TLRs in MDMs. MDMs were incubated with three different concentrations of α-defensins, 20, 500, and 1000nM overnight, and the expression levels of (A) TLR2 and (B) TLR4 were compared between MDM controls and α-defensin-treated MDMs. MDM controls and α-defensin-treated MDMs were infected with NTHi for three hours. (C) The expression level of TLR4 was compared among the four different samples. Statistical analysis was conducted using one-way ANOVA. Statistical significance is denoted by (*) (p-value < 0.05), and (ns) indicates no statistical difference among the samples. (D) The protein level of TLR4 was analyzed via SDS-PAGE. (E) The protein bands were quantified using NIH ImageJ software and compared between MDM controls and 1000nM of α-defensin-treated MDMs using a non-parametric Wilcoxon matched-pairs signed-rank test. Statistical significance is denoted by (*) (p-value < 0.05). [file Image3.tiff]
