# Supplementary material for: Alpha-defensins increase NTHi binding but not engulfment by the macrophages enhancing airway inflammation in Alpha-1 antitrypsin deficiency
Source: Front Immunol. 2025 Feb 12;16:1543729. doi: 10.3389/fimmu.2025.1543729 (PMC11861504; doi:10.3389/fimmu.2025.1543729)
Supplement: Supplementary file 4 [file Presentation1.pptx]

## Slide 1
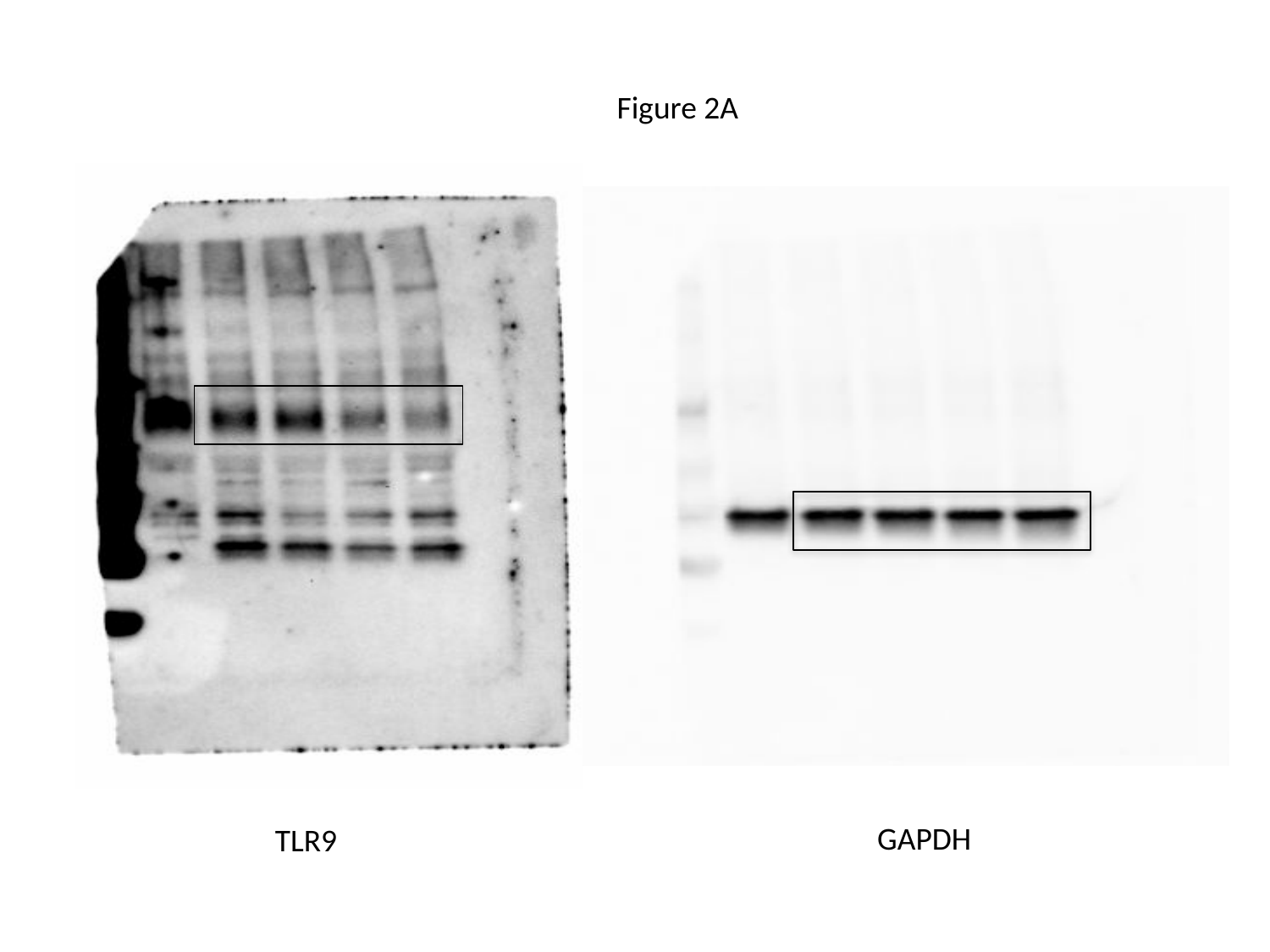

Figure 2A
GAPDH
TLR9

## Slide 2
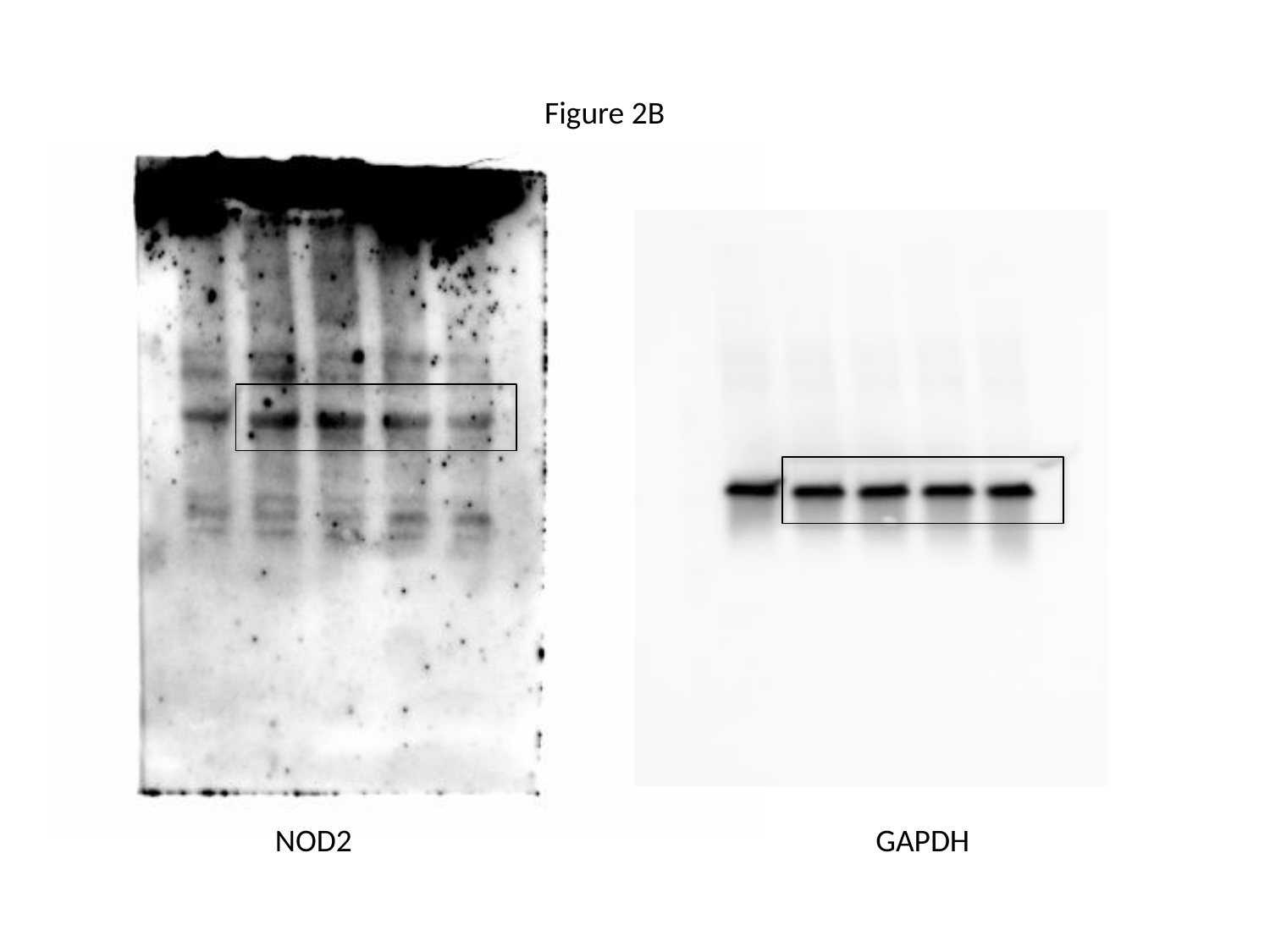

Figure 2B
 p-ERK1/2
 GAPDH
NOD2
GAPDH

## Slide 3
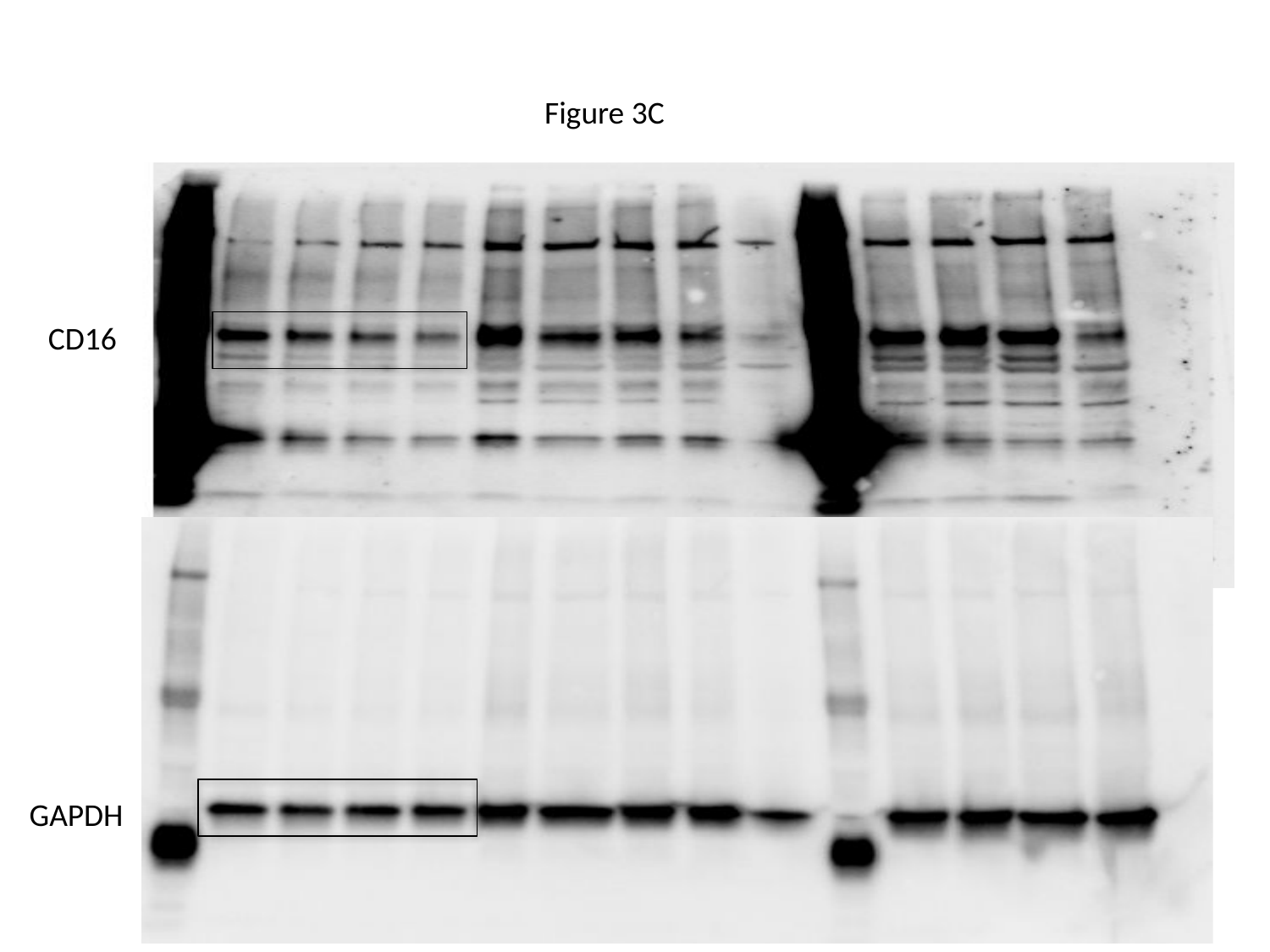

Figure 3C
CD16
GAPDH

## Slide 4
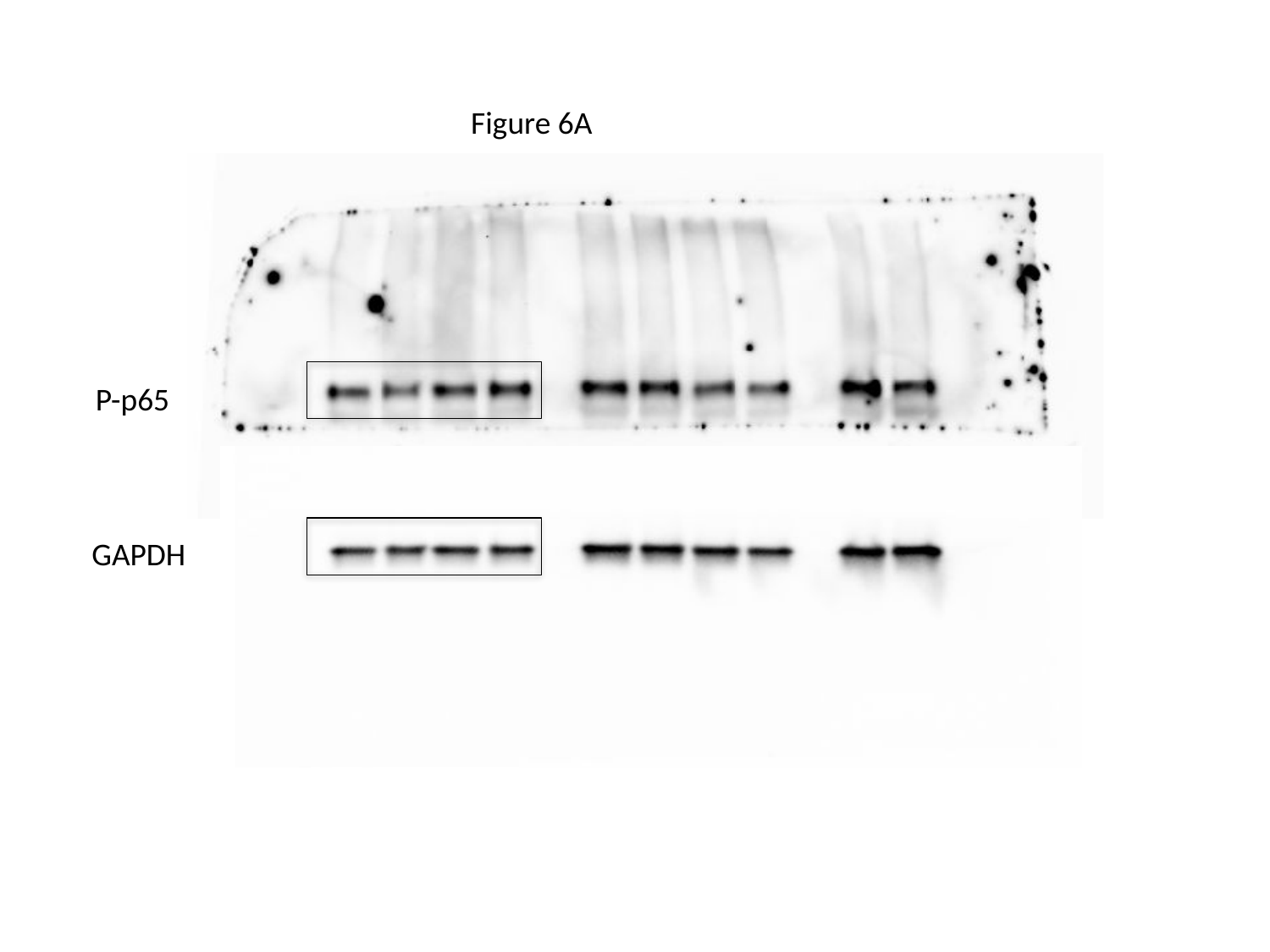

Figure 6A
P-p65
GAPDH

## Slide 5
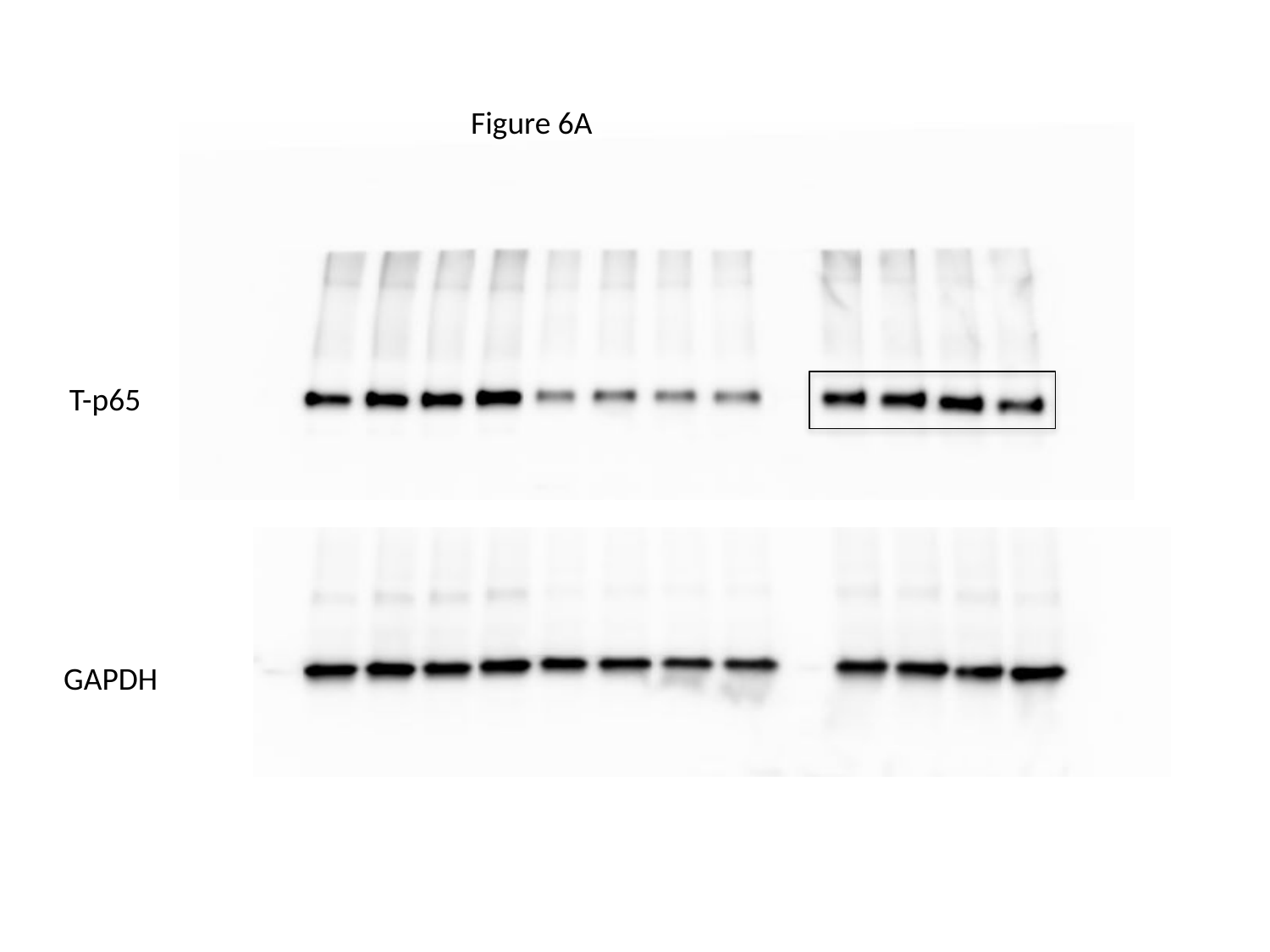

Figure 6A
T-p65
GAPDH

## Slide 6
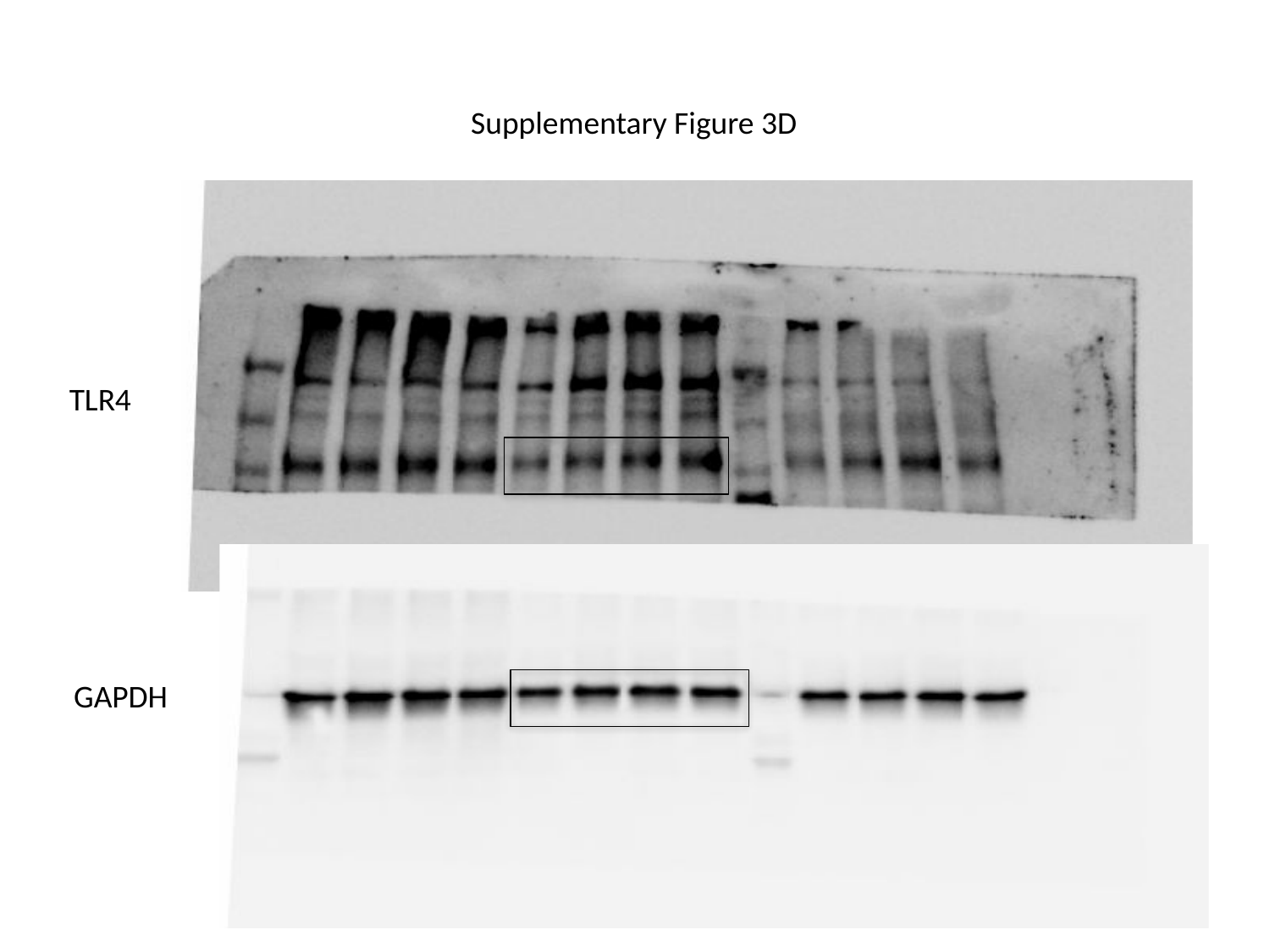

Supplementary Figure 3D
TLR4
GAPDH
